# Information Disclosure During the COVID-19 Epidemic in China: City-Level Observational Study

**DOI:** 10.2196/19572

**Published:** 2020-08-27

**Authors:** Guangyu Hu, Peiyi Li, Changzheng Yuan, Chenglin Tao, Hai Wen, Qiannan Liu, Wuqi Qiu

**Affiliations:** 1 Institute of Medical Information/Center for Health Policy and Management Chinese Academy of Medical Sciences and Peking Union Medical College Beijing China; 2 World Health Organization Collaborating Center for Health and Biomedical Information Beijing China; 3 Institute of Hospital Management West China Hospital of Sichuan University Chengdu China; 4 School of Public Health Zhejiang University Hangzhou China; 5 Shenzhen Medical Information Center Shenzhen China; 6 National Institute of Hospital Administration Beijing China

**Keywords:** information disclosure, COVID-19, website, risk, communication, China, disclosure, pandemic, health information, public health

## Abstract

**Background:**

Information disclosure is a top priority for official responses to the COVID-19 pandemic. The timely and standardized information published by authorities as a response to the crisis can better inform the public and enable better preparations for the pandemic; however, there is limited evidence of any systematic analyses of the disclosed epidemic information. This in turn has important implications for risk communication.

**Objective:**

This study aimed to describe and compare the officially released content regarding local epidemic situations as well as analyze the characteristics of information disclosure through local communication in major cities in China.

**Methods:**

The 31 capital cities in mainland China were included in this city-level observational study. Data were retrieved from local municipalities and health commission websites as of March 18, 2020. A checklist was employed as a rapid qualitative assessment tool to analyze the information disclosure performance of each city. Descriptive analyses and data visualizations were produced to present and compare the comparative performances of the cities.

**Results:**

In total, 29 of 31 cities (93.5%) established specific COVID-19 webpages to disclose information. Among them, 12 of the city webpages were added to their corresponding municipal websites. A majority of the cities (21/31, 67.7%) published their first cases of infection in a timely manner on the actual day of confirmation. Regarding the information disclosures highlighted on the websites, news updates from local media or press briefings were the most prevalent (28/29, 96.6%), followed by epidemic surveillance (25/29, 86.2%), and advice for the public (25/29, 86.2%). Clarifications of misinformation and frequently asked questions were largely overlooked as only 2 cities provided this valuable information. The median daily update frequency of epidemic surveillance summaries was 1.2 times per day (IQR 1.0-1.3 times), and the majority of these summaries (18/25, 72.0%) also provided detailed information regarding confirmed cases. The reporting of key indicators in the epidemic surveillance summaries, as well as critical facts included in the confirmed case reports, varied substantially between cities. In general, the best performance in terms of timely reporting and the transparency of information disclosures were observed in the municipalities directly administered by the central government compared to the other cities.

**Conclusions:**

Timely and effective efforts to disclose information related to the COVID-19 epidemic have been made in major cities in China. Continued improvements to local authority reporting will contribute to more effective public communication and efficient public health research responses. The development of protocols and the standardization of epidemic message templates—as well as the use of uniform operating procedures to provide regular information updates—should be prioritized to ensure a coordinated national response.

## Introduction

Prompt information disclosure is a top priority for preparedness and it enables a collective response to the COVID-19 pandemic [1]. In light of the important lessons learned from the global response to Ebola, reliable systems for sharing epidemiological and clinical data are essential for the timely production and dissemination of related knowledge [2]. Unfortunately, these systems had not been fully established before and during the COVID-19 outbreak in China. As the World Health Organization (WHO) declared the COVID-19 outbreak a public health emergency of international concern, people have the right to be clearly informed about the health risks that they and their communities face [3]. It is of great importance that authorities tackle the infodemic and improve response capacity, as they face a large amount of misinformation regarding the status of the pandemic. To help the general public and researchers bridge knowledge gaps and respond to the crisis in a timely manner, what information has been disclosed by the authorities was a crucial question. To address this issue, this study aimed to review and analyze the situation as it pertains to China.

Since the severe acute respiratory syndrome (SARS) emergency in 2003 and the avian influenza A (H7N9) epidemic in 2013, the Chinese government's provision of rapid, effective, and efficient disclosure of epidemic information has substantially improved [4,5]. Starting on January 3, 2020, information regarding COVID-19 cases has been reported to the WHO and the general public through China’s National Health Commission on a daily basis [6]. The time interval from the first case description to the identification of the pathogen on January 7—as well as the availability of probes for PCR detection on January 21—was much faster for COVID-19 than for SARS [7,8]. The National Health Commission took prompt public health measures and soon classified COVID-19 as a new notifiable disease under the National Infectious Disease Law and the Frontier Health and Quarantine Law on January 20, 2020, thus authorizing by law the prompt disclosure of information about the epidemic at the subnational level [9]. However, the lack of technical norms, standards, and actionable guidance from the national health authorities that could have served as a reference was the main issue thwarting efforts by local authorities to disclose information. Therefore, their main challenge was to determine the type and level of detail of essential information that could be disclosed as part of their COVID-19 epidemic responses.

In this article, we collectively reviewed the information disclosures related to the COVID-19 outbreak of 31 Chinese cities aggregated from online resources up to March 18, 2020. The data were recorded from the official websites of local municipalities or their health commissions. We described and compared the officially released content regarding the local epidemic situations and then analyzed the characteristics of information disclosure through local transmission for both the individual and total population levels in our attempt to present a detailed overview of information disclosure related to the COVID-19 epidemic in China.

## Methods

### Study Design

In this city-level observational study, our sample included all capital cities of the 31 provinces, autonomous regions, and municipalities in mainland China. There are 4 municipalities administered directly by the central government, 4 capital cities of ethnic minority autonomous regions, and 23 provincial capital cities. Generally, these cities are the socioeconomic and health care centers in their respective regions, and they have the highest population densities and the largest transregional migrant communities, all of which represent potentially higher risks of exposure to COVID-19 than smaller, less-dense neighboring cities.

We developed a checklist for assessing COVID-19 information disclosure, which could be used as a rapid qualitative assessment tool (Multimedia Appendix 1). It includes four sections: local summary report, content covered in the COVID-19 webpages, epidemic surveillance summary and confirmed case reports. We screened the relevant official public COVID-19 information from local municipalities and health commission websites and assessed each situation to allow participants to complete the checklist based on their observations. All authors independently reviewed the COVID-19 webpages of each city following a reviewer training process. The results of the checklists were cross-checked by the members of the research team. The corresponding author reviewed all webpages as well as the results of the final version. Discrepancies were resolved by discussion until consensus was reached. It must be noted that all of this information is publicly available. Patient consent was unnecessary, and no approvals from corresponding ethics boards were required.

### Data Sources

We queried the official websites of 31 sample cities to aggregate their information disclosure data related to the COVID-19 epidemic (Multimedia Appendix 2). As of March 18, 2020, there had been a total of 80,928 confirmed cases of COVID-19 on the Chinese mainland from the 31 provinces, autonomous regions, and municipalities. Meanwhile, no new domestically transmitted cases were reported for the first time since the outbreak [10]. This marked an important day that indicated that the intensity of the epidemic was diminishing and the increase in cases had slowed considerably as a result of effective national containment efforts. Therefore, we observed and reported records from the publicly available sources up to the date of this notable turning point.

Data collection was completed between March 25 and April 8, 2020. The city-level information disclosure records obtained using the checklist were formatted into a line-list database for further analysis, and screenshots of the corresponding webpages were archived for further reference (Multimedia Appendix 3).

### Outcome Measures

For the local summary report, the primary outcome was the number of cities that established specific public webpages for COVID-19 information. Cities were categorized as having provided a COVID-19 webpage if the local government or health commission published a webpage specifically for disclosure of information related to the local outbreak. Moreover, the secondary outcome was defined as the time interval from the date of the first confirmed case to the date of the release of this information.

For the content covered by the COVID-19 webpages, the primary outcome was the median number of content categories. We summarized a total of seven categories of prioritized information found on the front pages of the COVID-19 webpages and determined the distribution of these content categories.

For the epidemic surveillance summaries and the confirmed case reports, the primary outcome was the median frequency of daily updates of the epidemic surveillance summaries, and the secondary outcome was the median number of key facts disclosed in the case reports. We determined the specific indicators and related information recorded in the local epidemic situation updates either by March 18 or the latest date with available records.

### Statistical Analysis

We summarized the results using descriptive statistics. There are three types of administrative cities in China, each with its own administrative system; therefore we divided the included cities into three groups: the MC group (municipalities administered by the central government), the AC group (capitals of autonomous regions), and the PC group (provincial capitals). Categorical variables were summarized as counts and percentages. Numerical variables were reported as medians and interquartile ranges. No sampling weights were used because this was not a probabilistic sample. The analysis was performed using R (Version 3.4.3; R Foundation for Statistical Computing) and data visualizations were performed using Tableau Desktop (Version 2020.1.2; Tableau Software).

## Results

### Characteristics and Epidemic Summaries of the Cities

**Figure 1:**
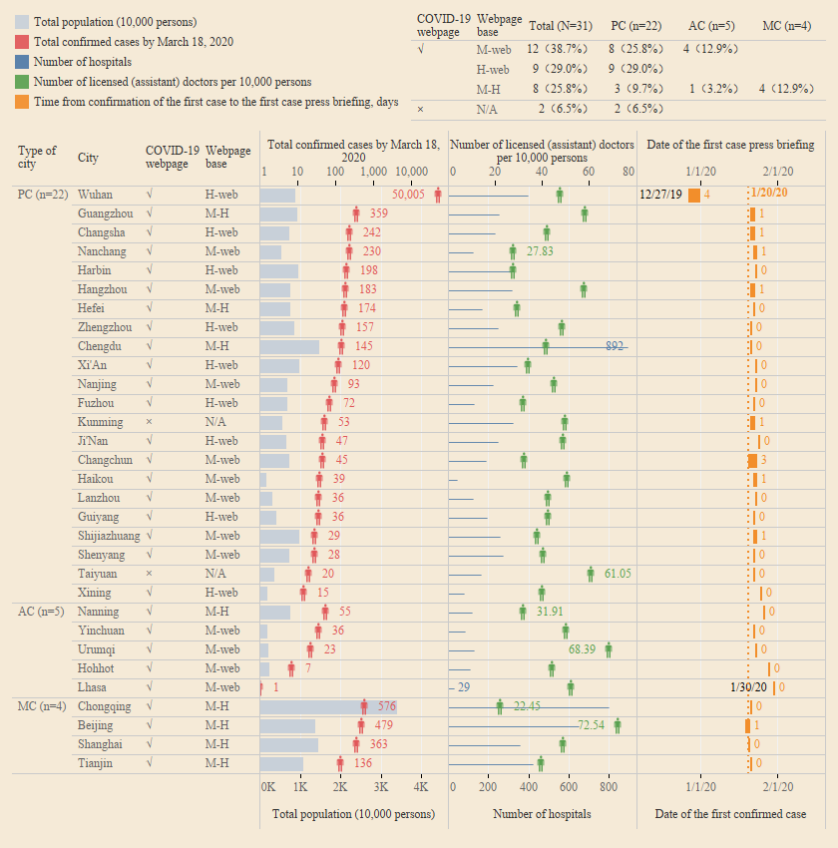
Characteristics of cities with confirmed COVID-19 cases included in this analysis. Data on total population, number of hospitals, number of licensed (assistant) doctors per 10,000 persons as of 2018 were obtained from the China Statistical Database. Chinese scientists identified the pathogen as a novel coronavirus on January 7, 2020. The first case with symptoms of the novel coronavirus in Wuhan was identified and reported to the authorities on December 27, 2019, by Jixian Zhang, the director of the Department of Respiratory and Critical Care Medicine of Hubei Provincial Hospital of Integrated Chinese and Western Medicine. China’s National Health Commission incorporated COVID-19 as a notifiable disease in the National Infectious Disease Law and the Frontier Health and Quarantine Law on January 20, 2020. AC: autonomous region capital; H-Web: health department website; MC: municipality administered by the central government; M-H: both municipality and health department websites; M-Web: municipality website; N/A: not applicable; PC: provincial capital.

We reviewed the records from the official websites of 31 cities. Of these, 29 (93.5%) created specific COVID-19 webpages to disclose information as follows: 12 webpages were added to the respective municipal websites, 9 were added to health department websites, and 8 were published on both the municipal and health department websites (Figure 1).

As of March 18, 2020, a total of 50,005 COVID-19 cases had been reported in Wuhan, a city in Hubei province, significantly more than all of the other sample cities. The number of licensed (assistant) doctors per 10,000 persons in Wuhan was 47.87, while the median for all 31 cites was 42.62 (IQR 36.18-50.09). The first case with novel coronavirus symptoms in Wuhan was identified and reported to authorities on December 27, 2019. Lhasa reported their first confirmed case on January 31, 2020, making the capital of the autonomous region of Tibet the last city to report a COVID-19 case. The median time from confirmation of the first case to the press briefing that publicly acknowledged that case was 0 days (ie, the same day; IQR 0-1). In fact, the majority of capital cities (21/31, 67.7%) publicly reported their first case of COVID-19 on the day of confirmation (Multimedia Appendix 4).

### Information Disclosure Highlights on Local COVID-19 Webpages

**Figure 2:**
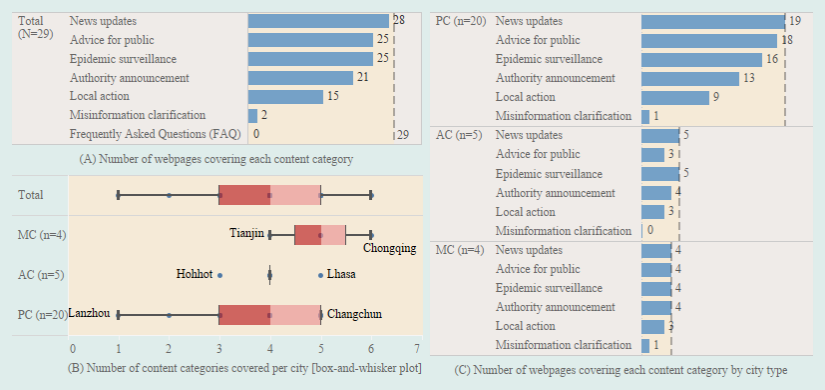
Description of content covered in the COVID-19 information webpages, March 2020. (A) Number of webpages covering each content category, summarized and identified from the cities’ COVID-19 webpages, as of March 2020. (B) Number of content categories covered per city; distribution was reported using a box-and-whisker plot and the blue dots with black text labels indicate the maximums and minimums. (C) Number of webpages covering each content category by city type, summarized and identified from the cities’ COVID-19 webpages, as of March 2020. AC: autonomous region capital; MC: municipality directly administered by the central government; PC: provincial capital.

We summarized the information disclosure highlights gathered from the local COVID-19 webpages of 29 capital cities and identified the categories of content covered in these webpages (Figure 2A). News updates were published by almost all of the cities (28/29, 96.6%). This category included the most recent news from local media and press briefings from several government sectors. Of the 29 cities, 25 (86.2%) released epidemic surveillance and advice for the public on their sites. Announcements from authorities were also commonly highlighted (21/29, 72.4%), followed by local actions (15/29, 51.7%) that included the authorities’ responses to local sporadic or widespread COVID-19 outbreaks. Only 2 of the 29 cities provided clarifications on previously published misinformation, and none of the cities provided lists of frequently asked questions.

Among the cites that developed COVID-19 webpages, the median number of content categories was 4 (IQR 3-5; Figure 2B). In terms of city type, the median for the MC group was higher than the other two groups at 5 (IQR 4-6), and the six categories on Chongqing’s webpage were the most of any city.

The percentage of content categories published on the COVID-19 webpages varied by city type (Figure 2C and Multimedia Appendix 5). The four municipalities administered by the Chinese central government all released at least four content categories on their official webpages as their responses to emergency information disclosure (Figure 2C). The disclosure performance of the provincial capitals and capitals of autonomous regions was worse compared with that of the centrally administered cities.

### Key Indicators Derived From the Epidemic Surveillance Summaries

**Figure 3:**
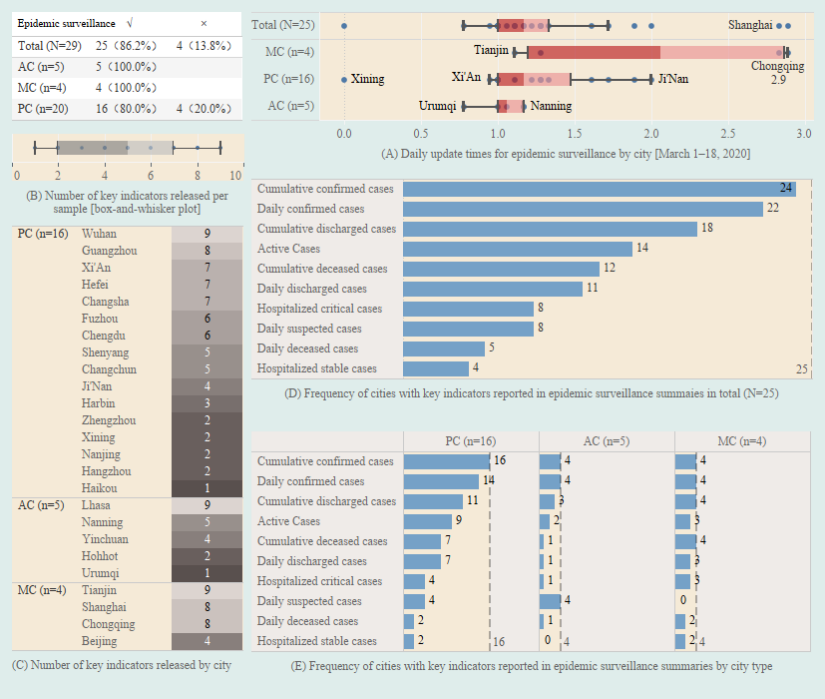
Key indicators reported in epidemic surveillance summaries as of March 18, 2020. (A) Daily update times for epidemic surveillance by city from March 1 to 18, 2020; distribution was reported using a box-and-whisker plot; blue dots with black text labels indicate maximums and minimums. (B) Number of key indicators released per sample; distribution was reported using a box-and-whisker plot. (C) Number of key indicators released by city; distribution was reported by heatmap. (D) Frequency of cities with key indicators reported in epidemic surveillance summaries in total (N=25). (E) Frequency of cities with key indicators reported in epidemic surveillance summaries by city type. AC: autonomous region capital; MC: municipality directly administered by the central government; PC: provincial capital.

Of the 29 cites with COVID-19 webpages, 25 (86.2%) highlighted epidemic surveillance summaries, while 4 (13.8%) neither disclosed local epidemic surveillance summaries nor provided local bulletins of the epidemic (Figure 3).

During the period from March 1 to 18, we calculated the daily update frequency for epidemic surveillance in each city from the records of the summaries (Figure 3A). The median daily update frequency was 1.2 times in total (IQR 1.0-1.3 times), while the MC group updated 2.1 times per day (IQR 1.2-2.9 times), the PC group 1.2 times (IQR 1.0-1.5 times), and the AC group 1.1 times (IQR 1.0-1.2 times).

We reviewed the local epidemic surveillance summaries of the 25 cities either on March 18, 2020, or the latest date with available records. The median number of key indicators reported in the summaries was 5 (IQR 2-7; Figure 3B). Wuhan, Lhasa, and Tianjin published at least nine key indicators in their summaries, and they were the cities with the most disclosed epidemic indicators in the PC, AC, and MC groups, respectively (Figure 3C).

The most common key indicators reported were the cumulative confirmed cases that appeared in the epidemic surveillance summaries of 24 cities (Figure 3D and Multimedia Appendix 6). Daily confirmed cases were published by 22 cities, cumulative discharged cases by 18, and active cases by 14. Less than half of the cities disclosed cumulative deceased cases (12/25, 48.0%) and daily discharged cases (11/25, 44.0%). Both hospitalized critical cases and daily suspected cases were reported in only 8 cities each (32.0%). Daily deceased cases and hospitalized cases of stable patients were released by 5 and 4 cites, respectively. A comparison of the frequency of key indicators reported by the cities reveals that, on average, the MC group released more indicators in their summaries than the others (Figure 3E).

### Details of the Confirmed Cases Reports

**Figure 4:**
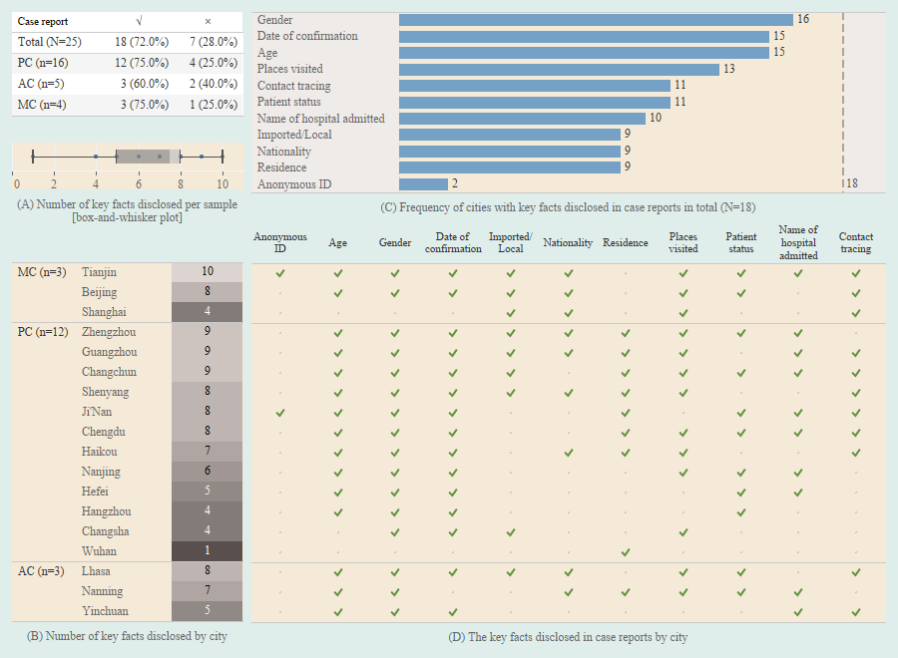
Key facts disclosed from the latest confirmed case reports as of March 18, 2020. (A) Number of key facts disclosed per sample; distribution was reported using a box-and-whisker plot. (B) Number of key facts disclosed by city; distribution was reported using a heatmap. (C) Frequency of cities with key facts disclosed in case reports in total (N=18). (D) The key facts disclosed in case reports by city. AC: autonomous region capital; MC: municipality directly administered by the central government; PC: provincial capital.

The majority of cities (18/25, 72.0%) provided detailed epidemic information of each confirmed case along with their epidemic surveillance summaries (Figure 4).

We identified the key facts disclosed in the detailed information about the latest confirmed cases as of March 18, 2020. The median number of key facts disclosed was 7.5 overall (IQR 5.0-8.0; Figure 4A); Tianjin disclosed the highest number of key facts at 10 and Wuhan the lowest at 1 (Figure 4B).

The genders of the patients with COVID-19 were disclosed in the case reports of most cities (16/18, 88.9%; Figure 4C). Dates of confirmation and age were usually included, with each being reported in 15 of the 18 cities (83.3%). Other information reported included the following: places visited (13/18, 72.2%), contact tracing (11/18, 61.1%), patient status (11/18, 61.1%), and the name of the hospital where each patient was admitted (10/18, 55.6%). Half of the cities that reported cases included whether the patient was a local resident or a migrant from somewhere else, their nationality, and their current residence. It is noteworthy that only two cities provided the anonymous ID of the confirmed patients per case (Figure 4D and Multimedia Appendix 7).

**Figure 5:**
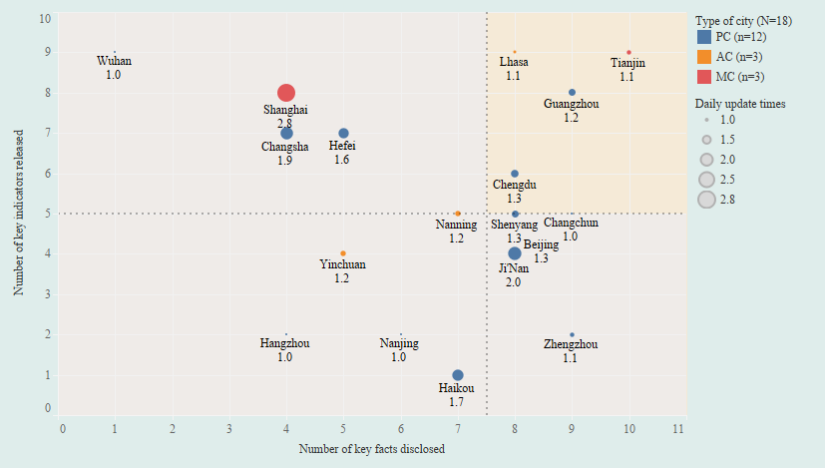
Scatterplots of the key facts disclosed in the case reports versus key indicators released in the epidemic surveillance summaries of 18 cities. The grey-shaded areas indicate cut-offs between the low number of key facts disclosed (lower than the median of 7.5) and the low number of key indicators released (lower than the median of 5.0). The dots representing Beijing and Ji’Nan coincide on the scatterplots. The name of the city and daily update frequencies for epidemic surveillance are displayed in black lettering.

The 4 cities with both the largest number of disclosed key facts and the largest number of reported key indicators are displayed in the orange-shaded areas of Figure 5. The number of key facts disclosed was similar to the number of key indicators released in these cities. However, a much greater disparity in the facts disclosed compared with the number of indicators released was noted for other cities, such as Wuhan and Zhengzhou. 

## Discussion

### Principal Results

This study presents the first summary of disclosure performance of local authorities related to COVID-19 epidemic information reporting in major mainland China cities as of March 2020. We found that most cities responded proactively and in a timely manner to disclose key information regarding the COVID-19 outbreak by publishing theme-based COVID-19 contents on the websites of local authorities. After the national authority incorporated COVID-19 as a notifiable disease by law on January 20, 2020, almost all the capital cities publicly reported their first case of COVID-19 on the day of confirmation or the following day via the official websites. News updates, epidemic surveillance, and advice for the general public have been the most frequently released contents on these COVID-19 webpages. The rapid and transparent reports published in China surpass the responses of most countries during the current pandemic [11].

We performed an assessment of the content released from the epidemic surveillance summaries and confirmed case reports of each city. There were variations in the key indicators released and key facts disclosed as part of the publicly available information recorded by each city in the study. Given the recent communication regarding COVID-19 risks and the community engagement action plan guidance that was developed and recommended by the WHO [3], we suggest that the significant dissimilarities in message and data templates for compiling epidemic surveillance summaries and confirmed case reports may need to be addressed in some of the cities as they have important implications for information disclosure and risk communication during any pandemic or serious event.

The general public’s misconceptions about COVID-19 in the United States and the United Kingdom in the early stages of the regional epidemic highlight the importance of timely and effective information disclosure by public health authorities [12]. This research revealed the efforts being made throughout the major municipalities of China. Regarding the scope of information released—as summarized and predefined in seven content categories—we revealed significant shortcomings in content related to misinformation clarification and frequently asked questions. Based on our review of the webpages archived in Multimedia Appendix 3 of this article, it is also worth noting that the information published on the webpages lacked clear content category labels for some cities (eg, Shijiazhuang and Lanzhou), and the content in some categories were mixed with unrelated material. As a resource that must provide prompt and accurate information, disorganization becomes an inevitable barrier for the dissemination of information to the general public. This finding underscores the need for clearly labeled content categories, proper sorting, and a clear focus on the needs of the audience when disclosing information during an emergency. In addition, the need for compliance with usability principles for information released on these types of websites has been highlighted in previous research published in the United States [13].

The epidemic surveillance summaries and confirmed case reports presented case-by-case in our research have been widely used as important resources for publicly available data in several clinical and epidemiological studies of COVID-19 [14-18]. As a valuable source of COVID-19 epidemiological data reported in the context of both entire populations and individuals, epidemic surveillance summaries usually include key indicators like COVID-19 cases, deaths, and recoveries, all of which may be used to track epidemic trends. A prime example is one of the most commonly cited web-based interactive dashboards [19], hosted by Johns Hopkins University [15], that has contributed information that supports public health decision making and global communication. In the present study, our results revealed dissimilarities between the key indicators released in the summary reports of different cities. Which kind of indicators should be reported to the public? There is still a lack of consensus on this topic among the municipal governments in China during the epidemic, as we have revealed in the analysis of indicators involved in the epidemic surveillance summaries. Owing to the lack of consistent protocols and standards used to report findings and data related to the epidemic, it is difficult for the general public to identify and interpret the entire scope and magnitude of the health risks they face. Meanwhile, if given properly presented information, professional researchers would have been able to collect and curate data as rapidly and widely as possible rather than compile it manually at higher labor costs because of incompatible record styles. Further, the numbers of daily hospital admissions and discharges, which are less-biased indicators for detecting changes in COVID-19 transmission dynamics [20], were not fully investigated in the epidemic summaries.

Concerning the confirmed case reports, although the machine-accessible, detailed, real-time, and robust individual-level epidemiology data for COVID-19 are publicly available [21], the primary data records used in the official case reports face the same challenge as the epidemic surveillance summaries as presented in our results. Another issue in the public reporting of individual confirmed patients on a case-by-case basis is the overall workload. With the epidemic continuing to expand quickly, this surge can surpass the response capacity of local authorities. At times, they have been unable to disclose information on an individual level as quickly as desired during the emergency. This was most notable in the case of Wuhan. Given that the details disclosed in the confirmed case reports were crucial to the estimation of key epidemiological parameters like incubation periods [17], severity [16], transmission dynamics [18], and prediction models [22], the responsibility of maintaining the integrity and impartiality of each recorded case was more critical than ever. However, upon our assessment of the details in the confirmed case reports, we observed a disparity in the contents and the number of indicators released by each municipality, and it is worth noting that almost all case reports from the local authorities could be improved for better accuracy and completeness to some extent. Moreover, rapid information disclosure and data sharing are necessary for informed public health decision making and subsequent actions during public health emergencies [23]. The balance between public information disclosure and individual privacy concerns must be addressed. Maintaining the confidentiality of patients’ names is challenging during a crisis like the COVID-19 pandemic. We found that anonymous identification was rarely used in the majority of confirmed case reports, while the surname, gender, age, and place of residence of confirmed cases were disclosed simultaneously in some cases (eg, Tianjin and Zhengzhou). The risk of personal information leaks in some cities’ case reports needs to be addressed, and further effort is needed to ensure the anonymity of individual patients.

### Limitations

There are several limitations to this study. First and foremost, capital cities are generally larger and denser, and they are unlikely to be representative of most Chinese cities. Therefore, the generalizability of our findings is limited. Second, the checklist we developed as a rapid qualitative assessment tool is somewhat subjective, and it is possible that some variations of information disclosure across the samples may be either overlooked or underestimated. Additional quantitative research would allow for a more accurate triangulation of the results. Third, local information disclosure performance might vary over time because of the rapidly evolving COVID-19 epidemiologic status in different regions. A retrospective longitudinal case study could further explain the evolution of the authorities’ responses during the outbreak. An examination of trust and feedback from the local population regarding the authorized sites would provide direct evidence for information communication improvement of the sites. Fourth, internet social media as another available major public resource was not included in this study. We assumed that the official websites of the cities in question would be the most authoritative sources of disclosed information. However, real-time and rolling COVID-19 updates available through social media channels have the potential to provide more information to the public. A more comprehensive future study could include social media as an alternative data source.

### Conclusions

Promoting the disclosure of information related to public health emergencies and providing the public with regular channels through which authoritative up-to-date information is disclosed are both essential for the timely communication of risk information and guidance. Our results augment the awareness of information and data disclosed on a city-by-city basis during the COVID-19 outbreak in China. The local authorities in major Chinese cities universally established COVID-19 webpages on their official websites to ensure the effective disclosure of epidemic information. Nevertheless, further improvements to local reporting practices will further contribute to effective public communication and more efficient public health research. This study offers insight into the deficiencies currently found in local information disclosure methods that were exposed during the COVID-19 epidemic in China. Therefore, the development of uniform protocols and standards of epidemic message templates must be encouraged, as should the use of standard operating procedures to regularly update all vital information in a manner that the public can easily interpret and researchers can effectively analyze. Our findings suggest that these issues should be considered a critical policy priority for the national health authorities in China and most countries worldwide.

## Appendix

Multimedia Appendix 1 COVID-19 information disclosure checklist for cities where one or more cases have been identified.

Multimedia Appendix 2 Data sources for the information disclosure records of the COVID-19 epidemic.

Multimedia Appendix 3 Screenshots of the front page of the COVID-19 webpages.

Multimedia Appendix 4 Characteristics of cities with confirmed COVID-19 cases included in this analysis.

Multimedia Appendix 5 Percentage of cities with content categories covered on official COVID-19 websites, March 2020.

Multimedia Appendix 6 Percentage of cities with key indicators revealed in epidemic surveillance summaries, as of March 18, 2020.

Multimedia Appendix 7 Percentage of cities with key facts disclosed from the latest confirmed case reports, as of March 18, 2020.

## Abbreviations

AC: capitals of autonomous regions
H7N9: avian influenza A
MC: municipalities administered by the central government
PC: provincial capitals
SARS: severe acute respiratory syndrome
