# Supplementary material for: Information Disclosure During the COVID-19 Epidemic in China: City-Level Observational Study
Source: J Med Internet Res. 2020 Aug 27;22(8):e19572. doi: 10.2196/19572 (PMC7473703; doi:10.2196/19572)
Supplement: Multimedia Appendix 1 [file jmir_v22i8e19572_app1.docx]

| **COVID-19 information disclosure checklist for cities where one or more cases have been identified** | |
| --- | --- |
| This tool, which is similar to the questions asked in qualitative form could be used as a rapid qualitative assessment tool to analyze city-level information disclosure during the COVID-19 epidemic. | |
| **Local summary**  Note: Please describe characteristics and the current local epidemic situation of the city | |
| City: |  |
| Type of city | |
| ‌‍ | Provincial capital |
| ‌‍ | Autonomous region capital |
| ‌‍ | Municipality administered by the central government |
| Total Population (10000 persons): | |
| Number of Hospitals: | |
| Number of Licensed (Assistant)Doctors per 10000 Persons: | |
| Total confirmed cases by 18 March 2020: | |
| Date of the first confirmed case: | |
| Date of the first case press briefing: | |
| COVID-19 webpage based on | |
| ‌‍ | Not applicable (No specific COVID-19 webpage) |
| ‌‍ | Municipality website |
| ‌‍ | Health department website |
| **Content covered in the COVID-19 webpages**  Note: Please mark all those highlights you have seen on the webpages. | |
| ‌‍ | News updates |
| ‌‍ | Authority announcement |
| ‌‍ | Epidemic surveillance |
| ‌‍ | Local action |
| ‌‍ | Advice for public |
| ‌‍ | Misinformation clarification |
| ‌‍ | Frequently Asked Questions (FAQs) |
| **Key indicators reported in epidemic surveillance summary**  Note: Please mark all those indicators reported in the daily epidemic surveillance summary. | |
| ‌‍ | Cumulative confirmed cases |
| ‌‍ | Cumulative discharged cases |
| ‌‍ | Cumulative deceased cases |
| ‌‍ | Daily confirmed cases |
| ‌‍ | Daily suspected cases |
| ‌‍ | Daily discharged cases |
| ‌‍ | Daily deceased cases |
| ‌‍ | Active Cases |
| ‌‍ | Hospitalized stable cases |
| ‌‍ | Hospitalized critical cases |
| ‌‍ | Epidemic curve |
| **Key facts disclosed from the latest confirmed case report**  Note: Please mark all facts disclosed from the case report as of March 18, 2020. | |
| ‌‍ | Case identification by anonymous code |
| ‌‍ | Age |
| ‌‍ | Gender |
| ‌‍ | Date of confirmation |
| ‌‍ | Imported/Local |
| ‌‍ | Nationality |
| ‌‍ | Residence |
| ‌‍ | Places visited |
| ‌‍ | Patient status |
| ‌‍ | Name of hospital admitted |
| ‌‍ | Contact tracing |
