# Supplementary material for: Information Disclosure During the COVID-19 Epidemic in China: City-Level Observational Study
Source: J Med Internet Res. 2020 Aug 27;22(8):e19572. doi: 10.2196/19572 (PMC7473703; doi:10.2196/19572)
Supplement: Multimedia Appendix 2 [file jmir_v22i8e19572_app2.docx]

**Multimedia Appendix 2. Data sources for the information disclosure records of the COVID-19 epidemic**

| **City** | **Webpage base** | **Main sources for extracting information disclosure record** |
| --- | --- | --- |
| Beijing | Municipality website  Health department website | [http://wjw.beijing.gov.cn/wjwh/ztzl/xxgzbd](http://wjw.beijing.gov.cn/wjwh/ztzl/xxgzbd/)  http://www.beijing.gov.cn/ywdt/zwzt/yqfk |
| Tianjin | Municipality website  Health department website | <http://www.tj.gov.cn/xw/ztzl/tjsyqfk>  <http://wsjk.tj.gov.cn/col/col86/index.html> |
| Shijiazhuang | Municipality website | http://www.sjz.gov.cn/col/1580262555218/index.html |
| Taiyuan | N/A | N/A |
| Hohhot | Municipality website | http://www.huhhot.gov.cn/ztzl/yqfk |
| Shenyang | Municipality website | http://www.shenyang.gov.cn/syszf/zt/fkxx/glist.html |
| Changchun | Municipality website | http://www.changchun.gov.cn/ztlm/qlkjxxgzbdgrdfyyqcczxd |
| Harbin | Health department website (CDC) | https://hrbcdc.jkhrb.org.cn/listOnePage.do?ID=10284a324095496d9707c8160776accd |
| Shanghai | Municipality website  Health department website | [http://wsjkw.sh.gov.cn/yqfk2020](http://wsjkw.sh.gov.cn/yqfk2020/)  http://www.shanghai.gov.cn/nw2/nw2314/nw32419/nw48516/index.html |
| Nanjing | Municipality website | http://www.nanjing.gov.cn/zt/yqfk/index.html |
| Hangzhou | Municipality website | http://www.hangzhou.gov.cn/col/col1228998456/index.html |
| Hefei | Municipality website  Health department website | <http://wjw.hefei.gov.cn/ztzl/xxgzbdgrdfyyqfk/index.html>  <http://www.hefei.gov.cn/ssxw/ztzl/zt/fkxxgzbdgrfyyqzxd/index.html> |
| Fuzhou | Health department website | <http://www.fuzhou.gov.cn/zgfzzt/swjw/fzwj/qlzhxxgzbdfyyqfkgz/> |
| Nanchang | Municipality website | http://www.nc.gov.cn/qlzhyqfk/index.shtml |
| Ji'Nan | Health department website | http://jnmhc.jinan.gov.cn/col/col50360/index.html |
| Zhengzhou | Health department website | http://wjw.zhengzhou.gov.cn/qwfb/index.jhtml |
| Wuhan | Health department website | http://wjw.wuhan.gov.cn/front/web/ztzl/801 |
| Changsha | Health department website | http://wsjkw.changsha.gov.cn/ztzl_1/fkxxgzbd/index.html |
| Guangzhou | Municipality website  Health department website | <http://www.gz.gov.cn/zt/qlyfdyyqfkyz/index.html>  http://wjw.gz.gov.cn/ztzl/xxfyyqfk |
| Nanning | Municipality website  Health department website | <http://www.nanning.gov.cn/zt/2018nztlm/xxgzbd>  http://wjw.nanning.gov.cn/ztjj/xxgzbd/index.html |
| Haikou | Municipality website | http://www.haikou.gov.cn/zfdt/ztbd/2020ztbd/dyyqfkzjz |
| Chongqing | Municipality website  Health department website | <http://www.cq.gov.cn/yqzl>  http://www.wsjkw.cq.gov.cn/ztzl_242/qlzhxxgzbdfyyqfkgz |
| Chengdu | Municipality website  Health department website | <http://www.chengdu.gov.cn/chengdu/c135620/cdyqzjz.shtml>  http://cdwjw.chengdu.gov.cn/cdwjw/c135632/yqbd.shtml |
| Guiyang | Health department website | http://wsjkj.guiyang.gov.cn/xxgzbdgrfyfk/tzgg |
| Kunming | N/A | N/A |
| Lhasa | Municipality website | http://www.lasa.gov.cn/lasa/c101149/lsfyzt.shtml |
| Xi'An | Health department website | http://xawjw.xa.gov.cn/ztzl/fyfk/gzdt/1.html |
| Lanzhou | Municipality website | http://www.lanzhou.gov.cn/col/col14903/index.html |
| Xining | Health department website | https://wsjkw.qinghai.gov.cn/ztbd/yqjk/index.html |
| Yinchuan | Municipality website | http://www.yinchuan.gov.cn/zhuanti/wzyxkjyq/index.html |
| Urumqi | Municipality website | http://www.urumqi.gov.cn/info/iIndex.jsp?cat_id=16159 |
