# Supplementary material for: Information Disclosure During the COVID-19 Epidemic in China: City-Level Observational Study
Source: J Med Internet Res. 2020 Aug 27;22(8):e19572. doi: 10.2196/19572 (PMC7473703; doi:10.2196/19572)
Supplement: Multimedia Appendix 3 [file jmir_v22i8e19572_app3.doc]

**Appendix 3. Screenshots of the front page of the COVID-19 webpages**

- **Beijing-Municipality website**

**
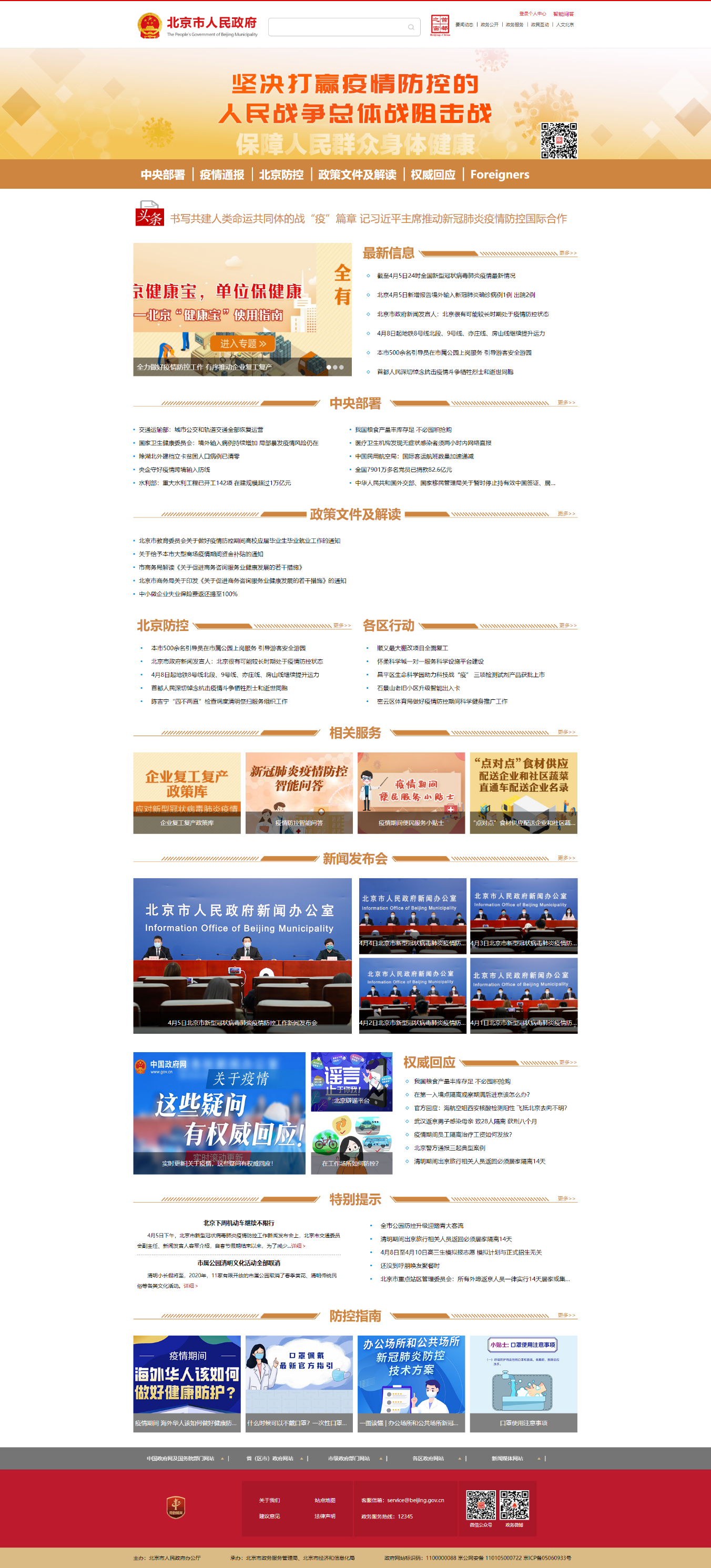
**

- **Beijing
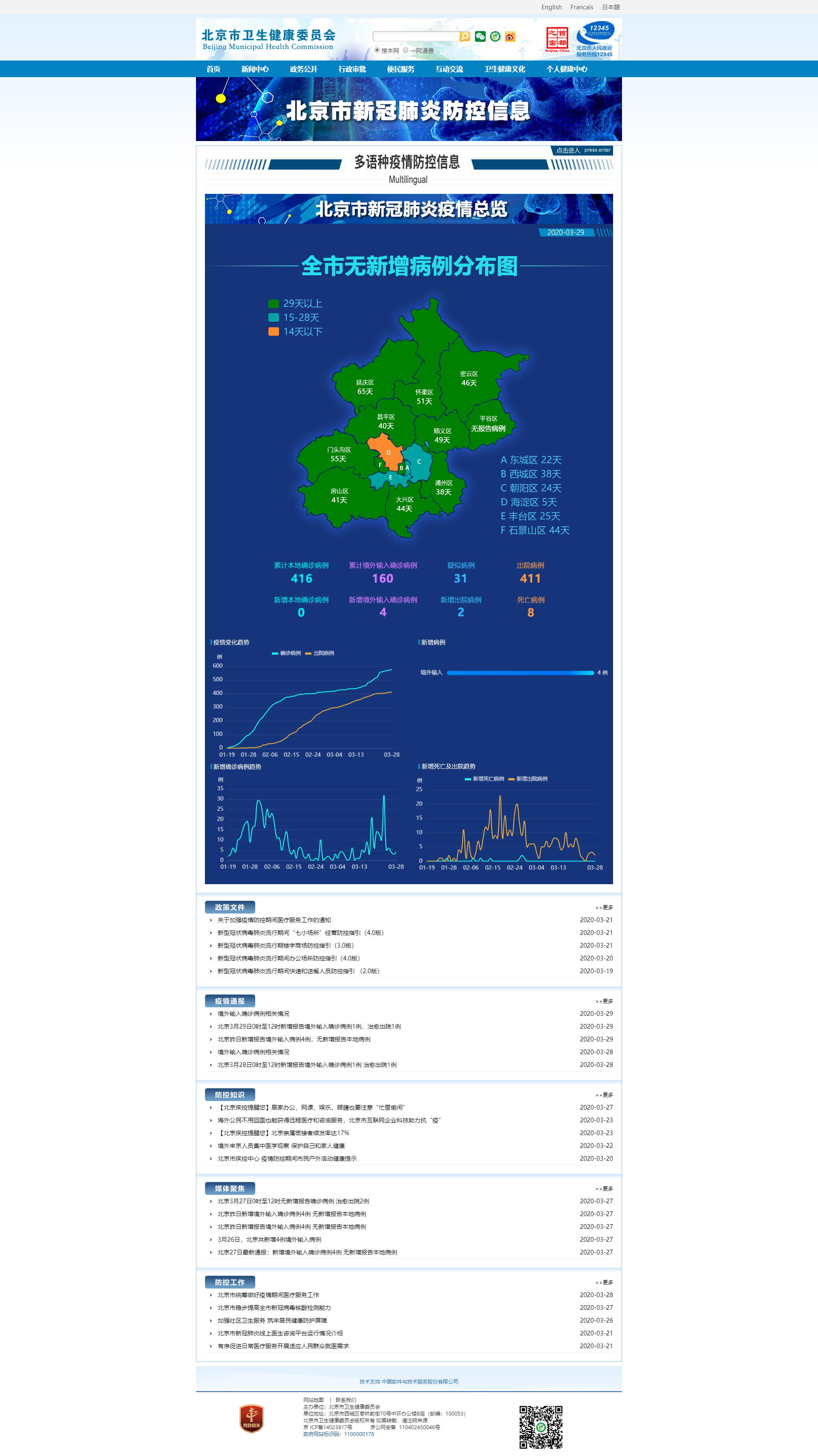
-Health department website**
- **Tianjin-Municipality website**

**
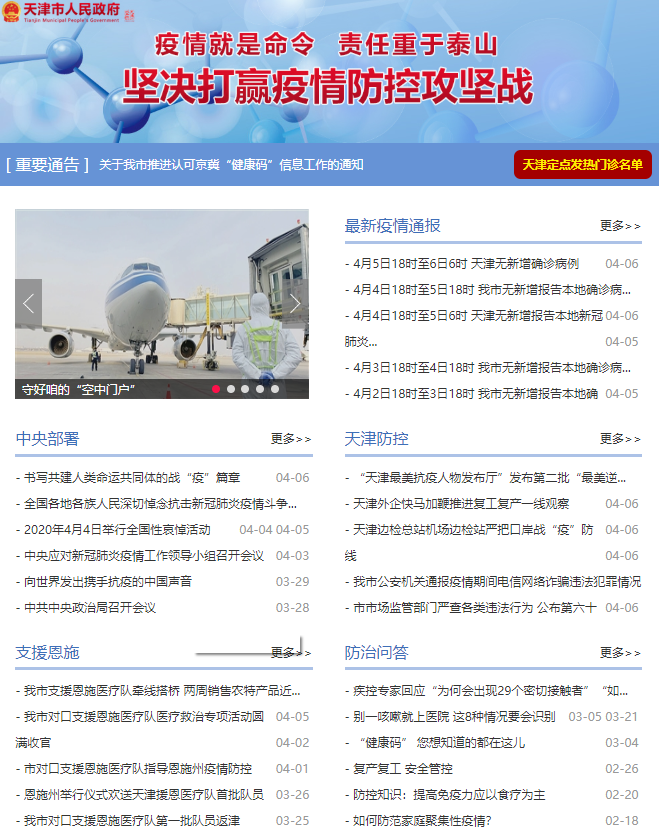
**

- **Tianjin-Health department website**


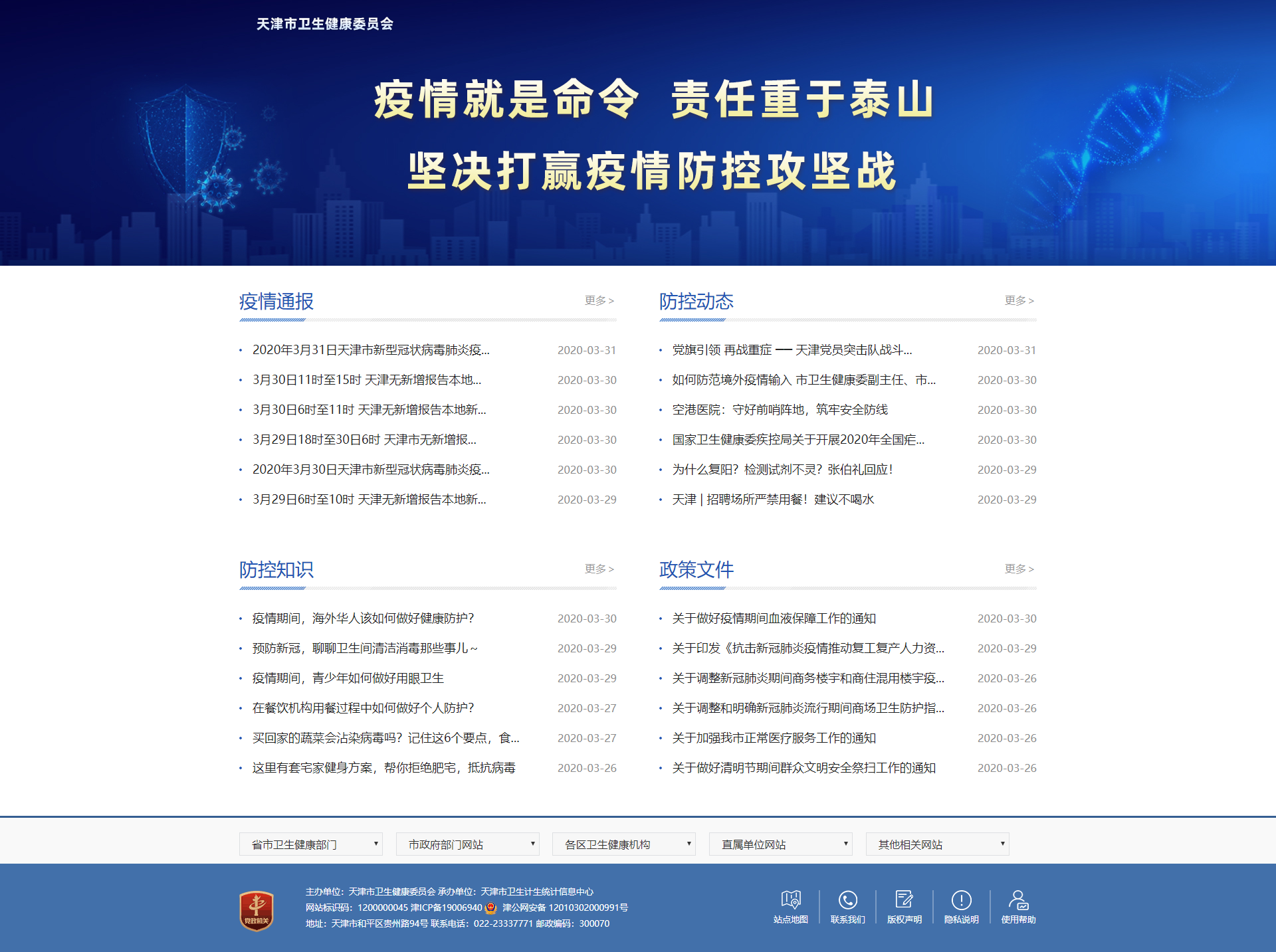


- **Shijiazhuang-Municipality website**

**
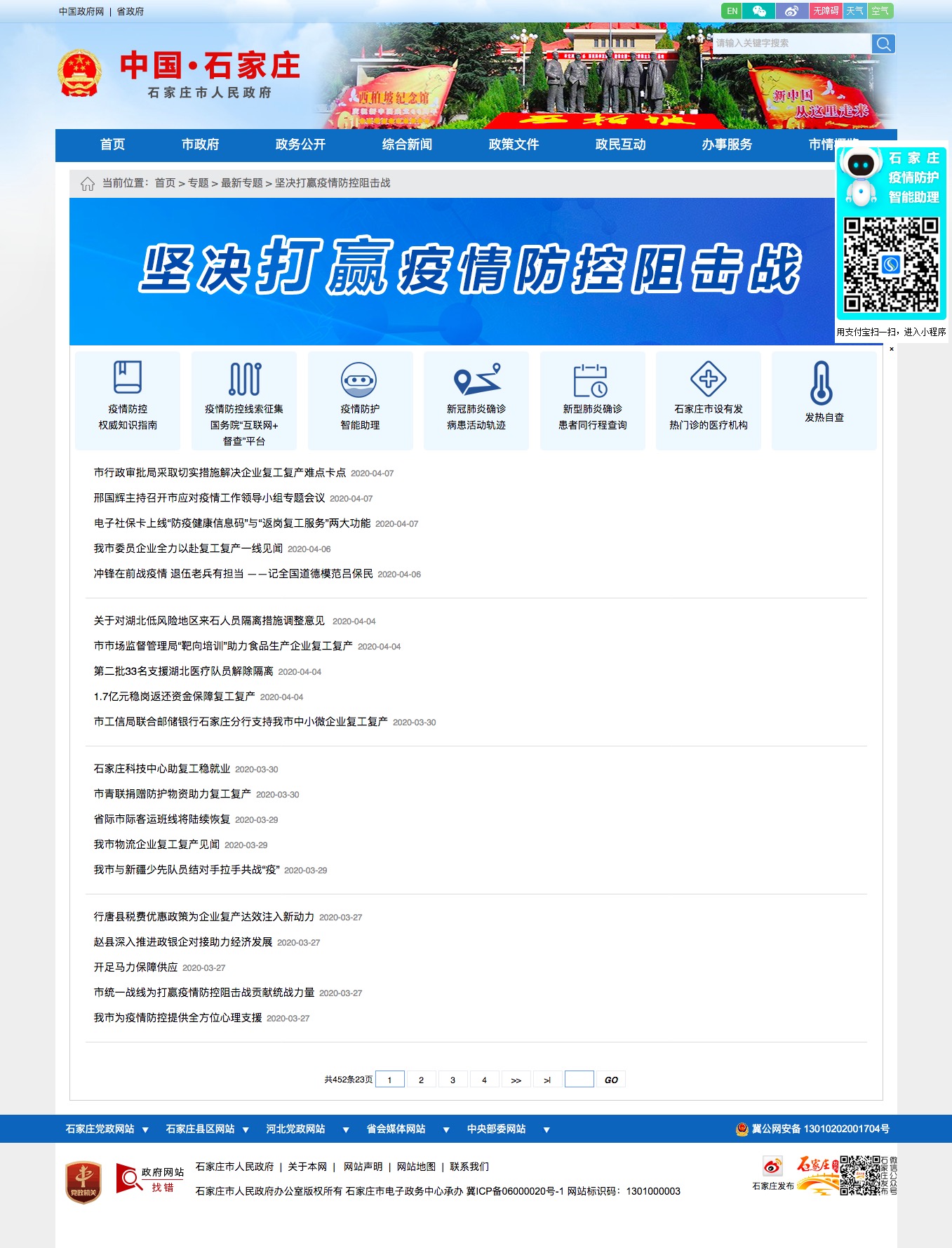
**

- **Hohhot-Municipality website**

**
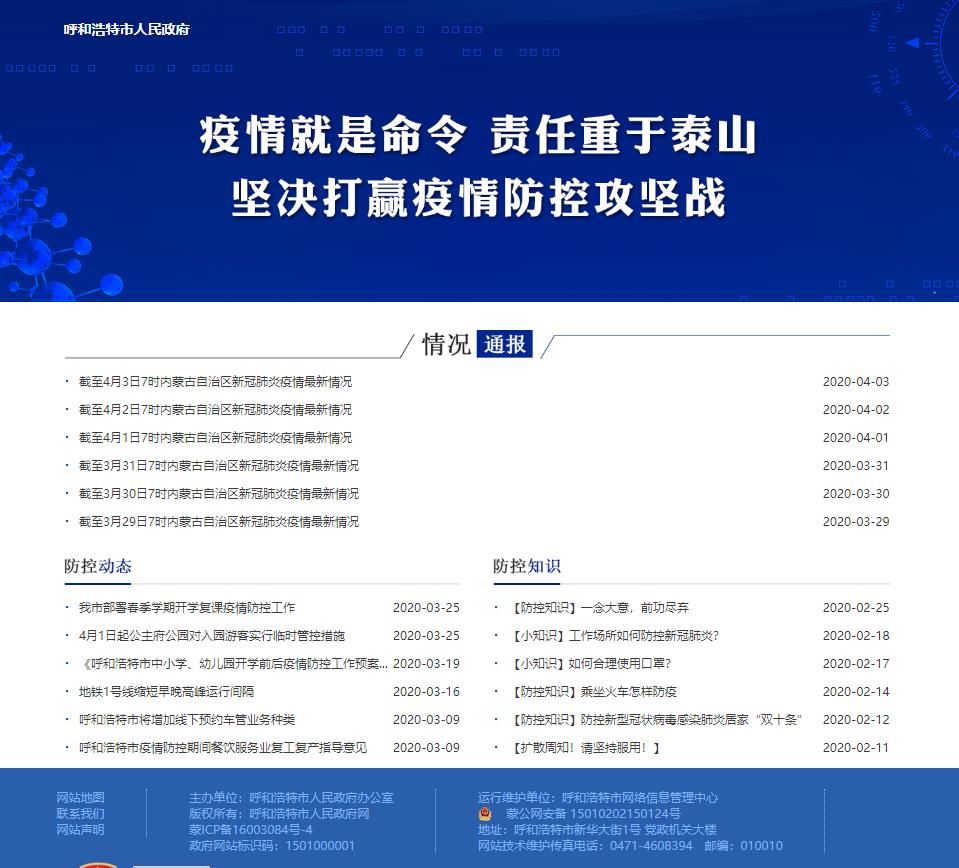
**

- **Shenyang-Municipality website**

**
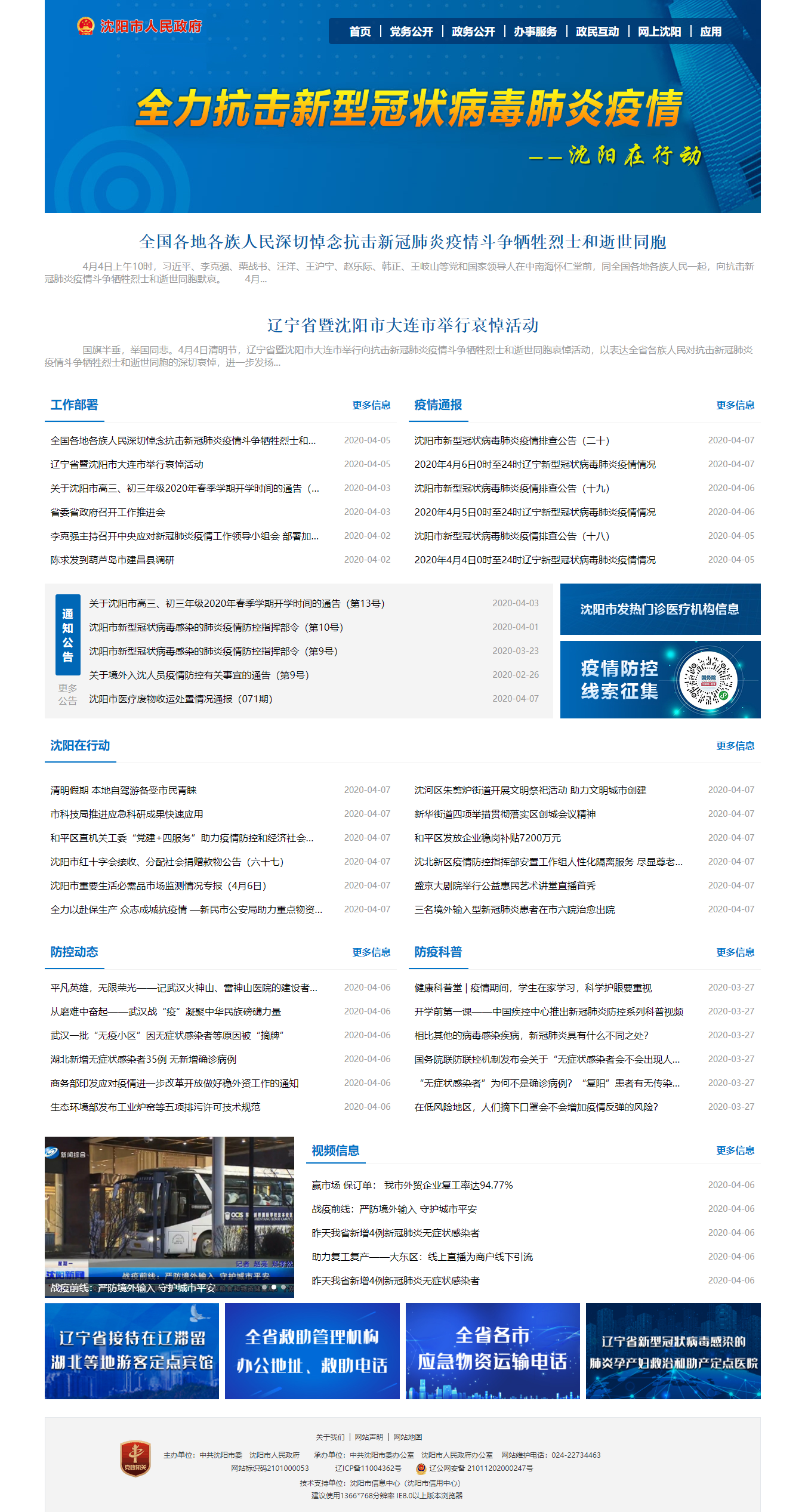
**

- **Changchun-Municipality website**

**
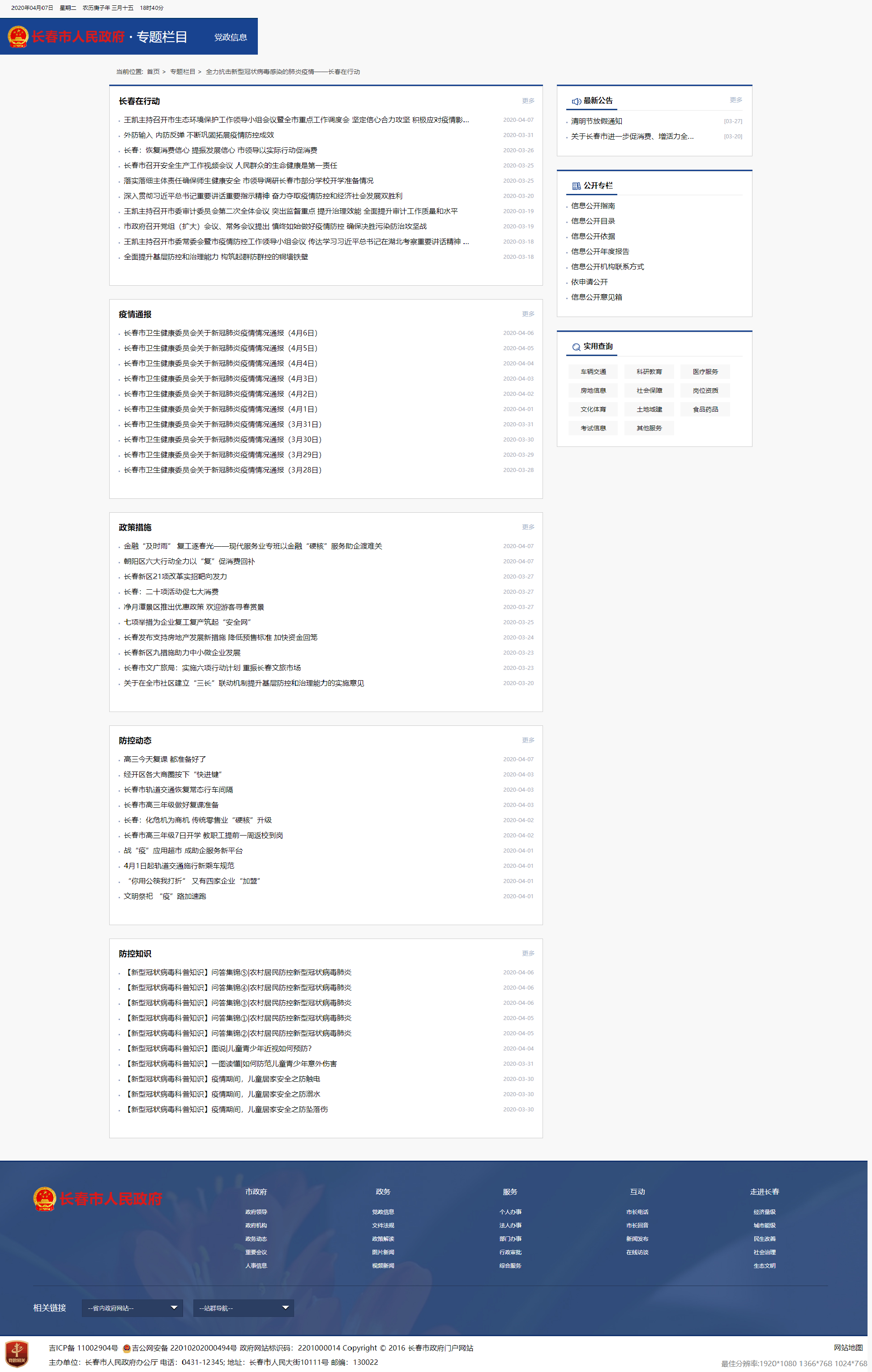
**

- **Harbin-Health department website (CDC)**

**
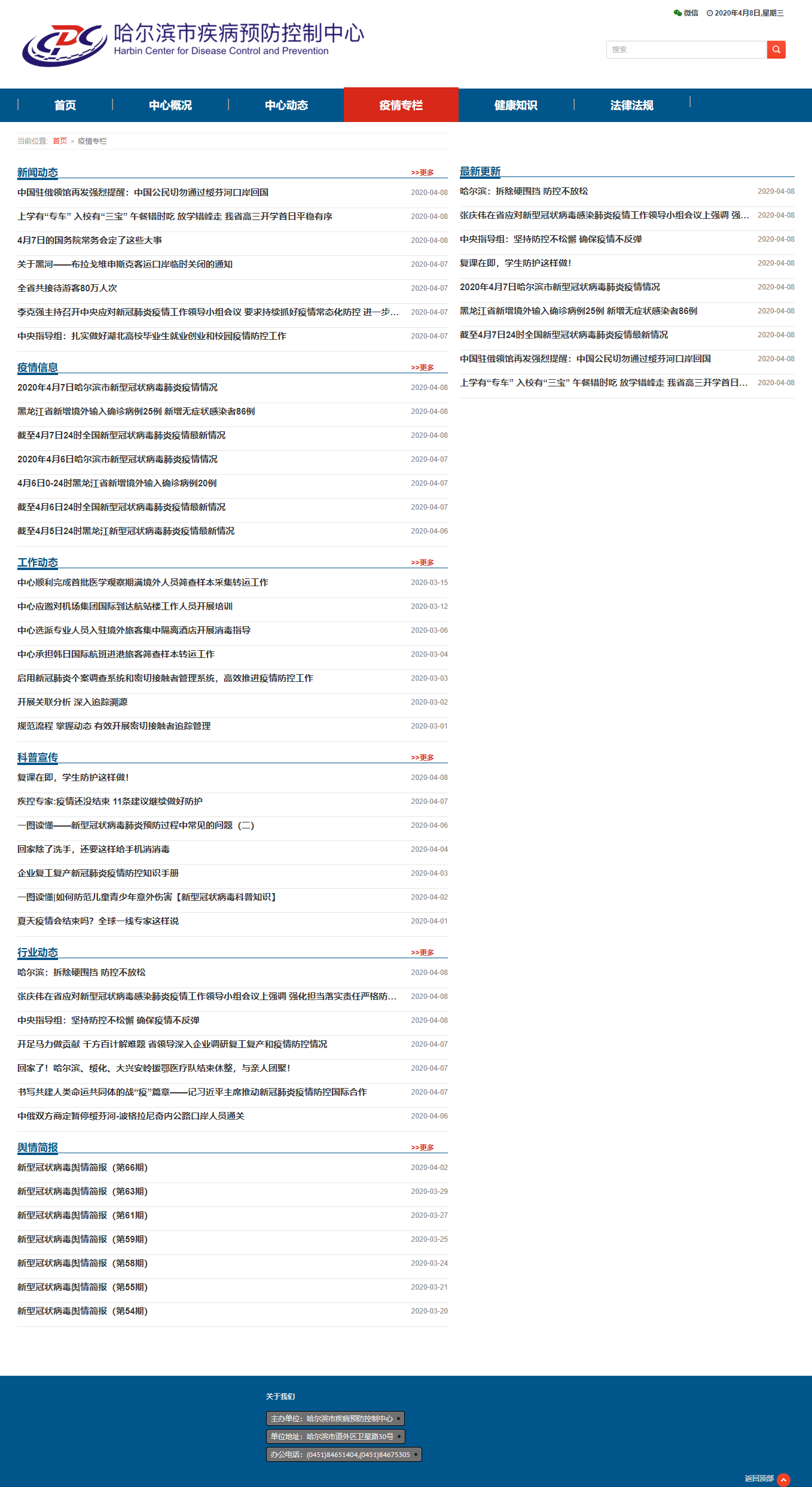
**

- **Shanghai-Municipality website**

**
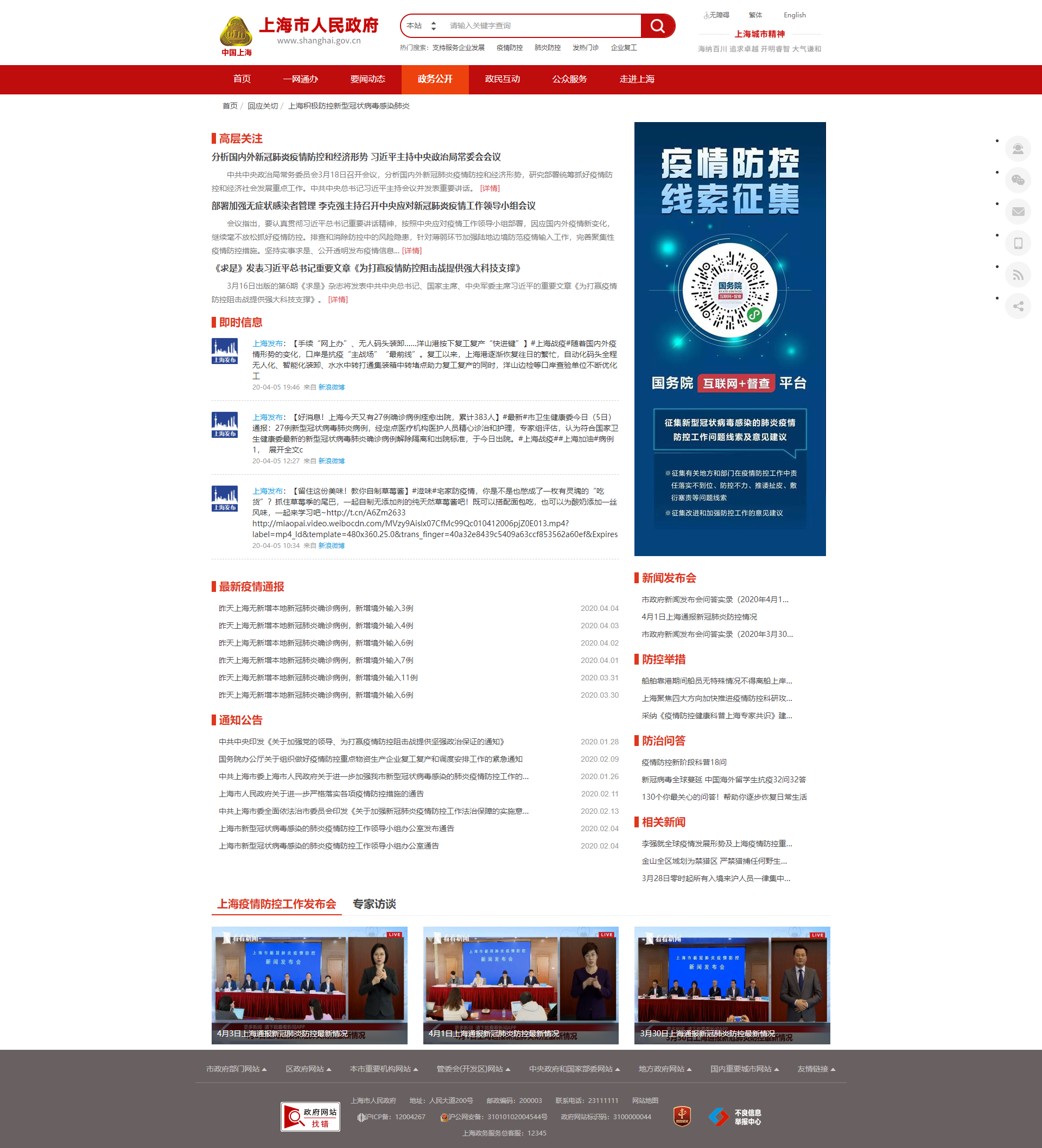
**

- **Shanghai-Health department website**

**
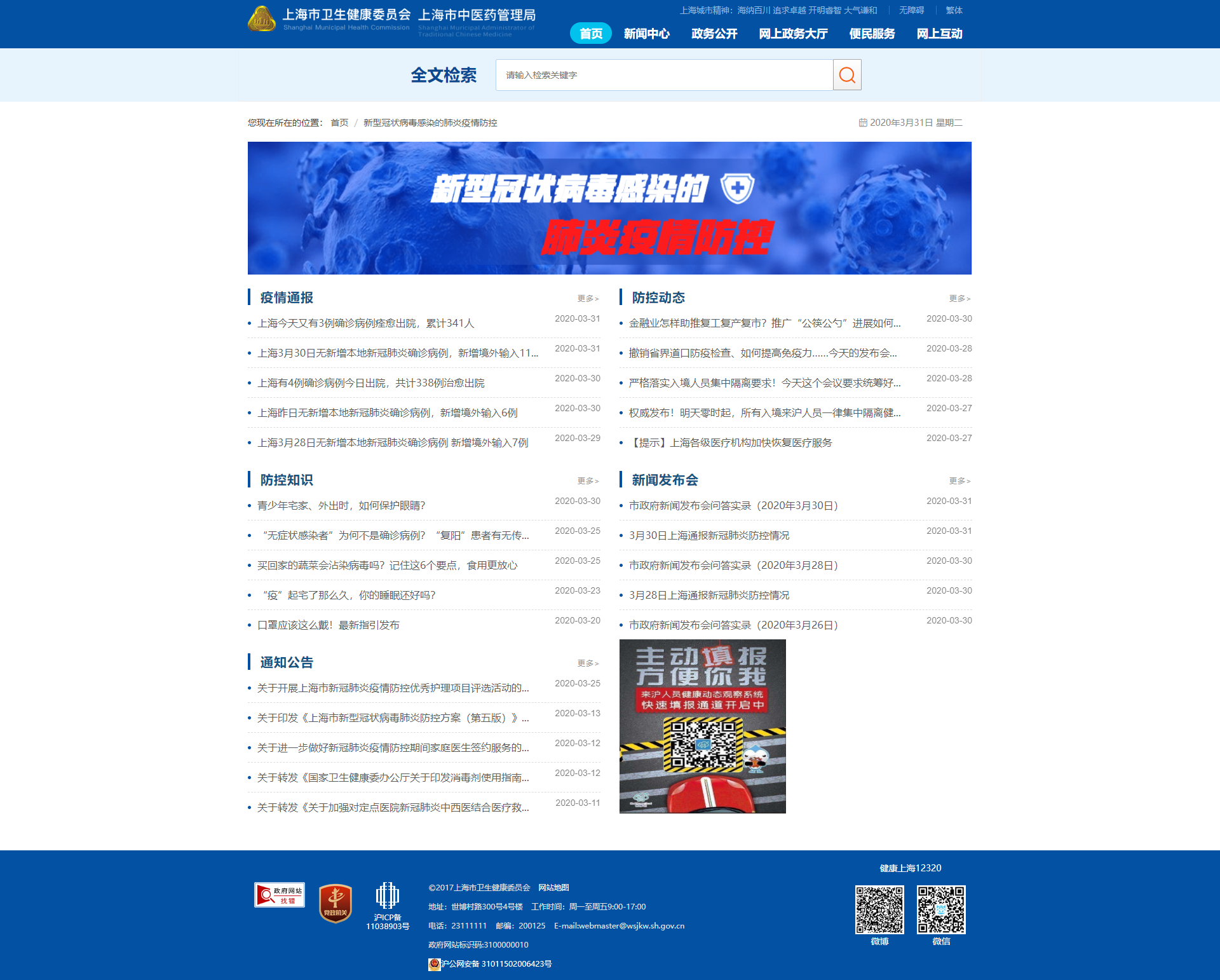
**

- **Nanjing-Municipality website**


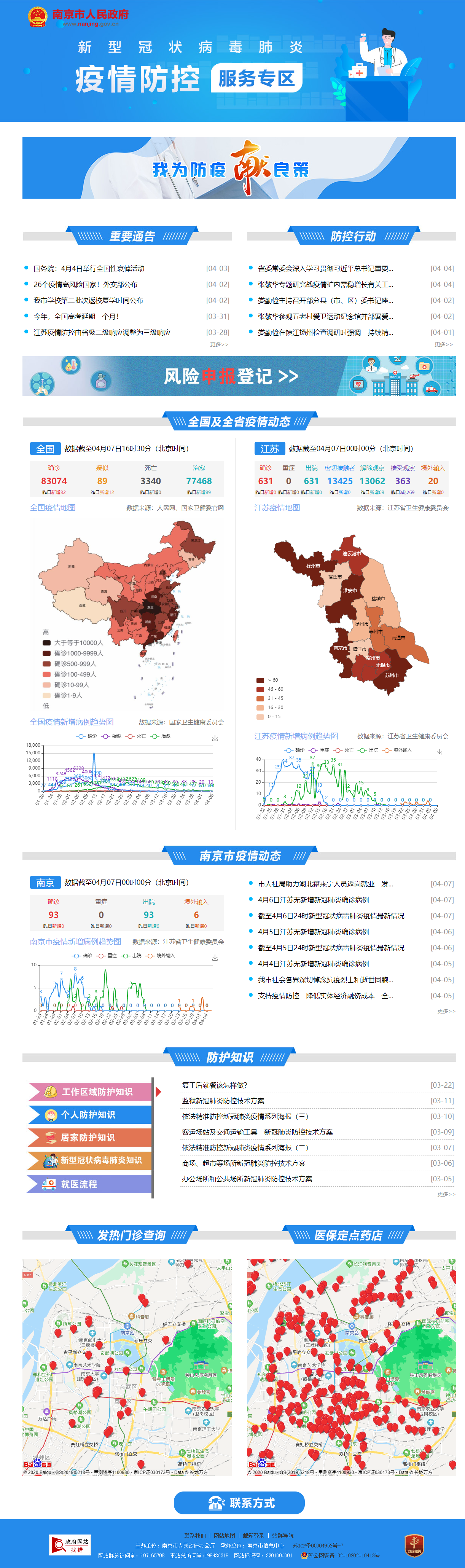


- **Hangzhou-Municipality website**

**
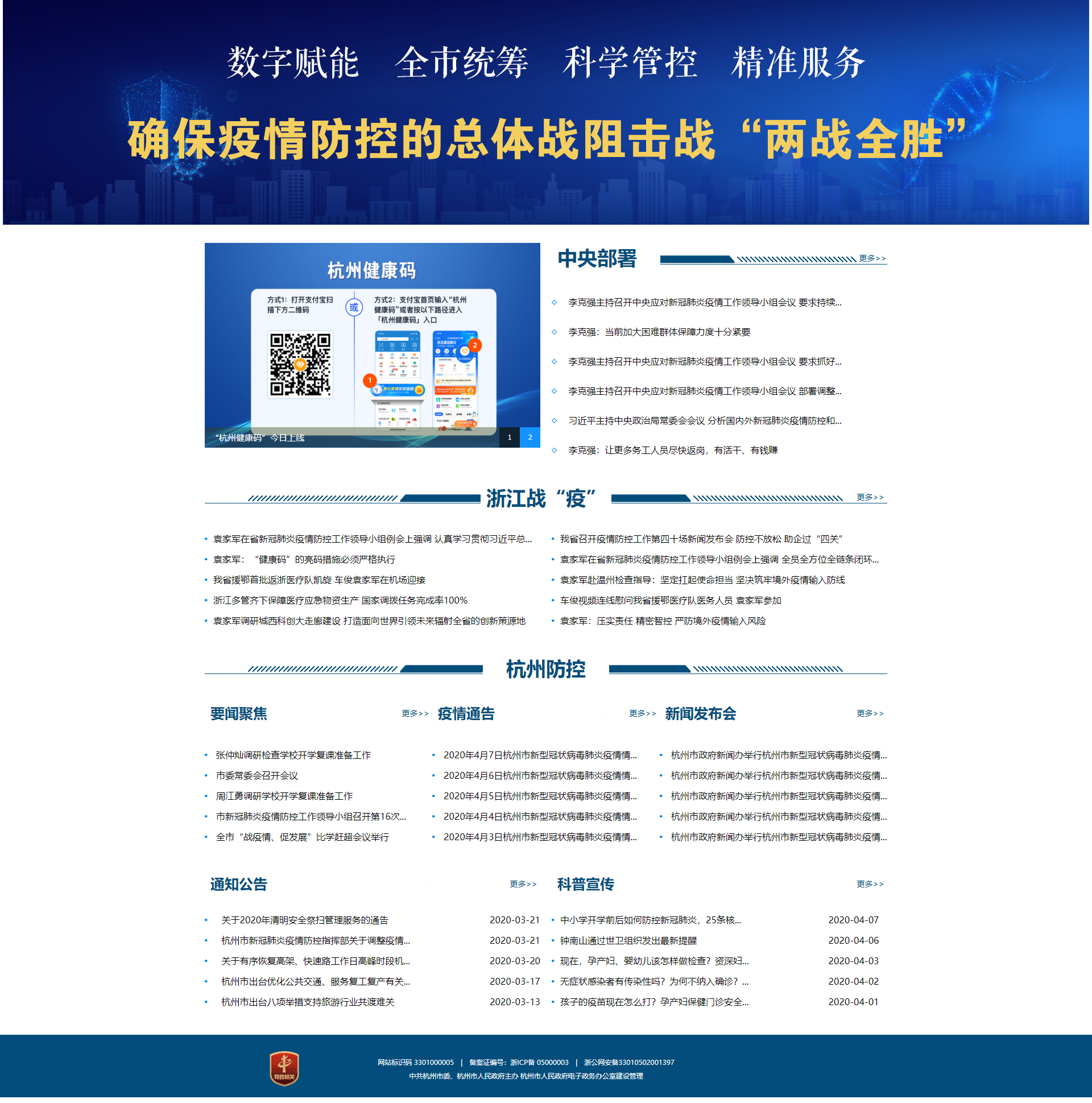
**

- **Hefei-Municipality website**

**
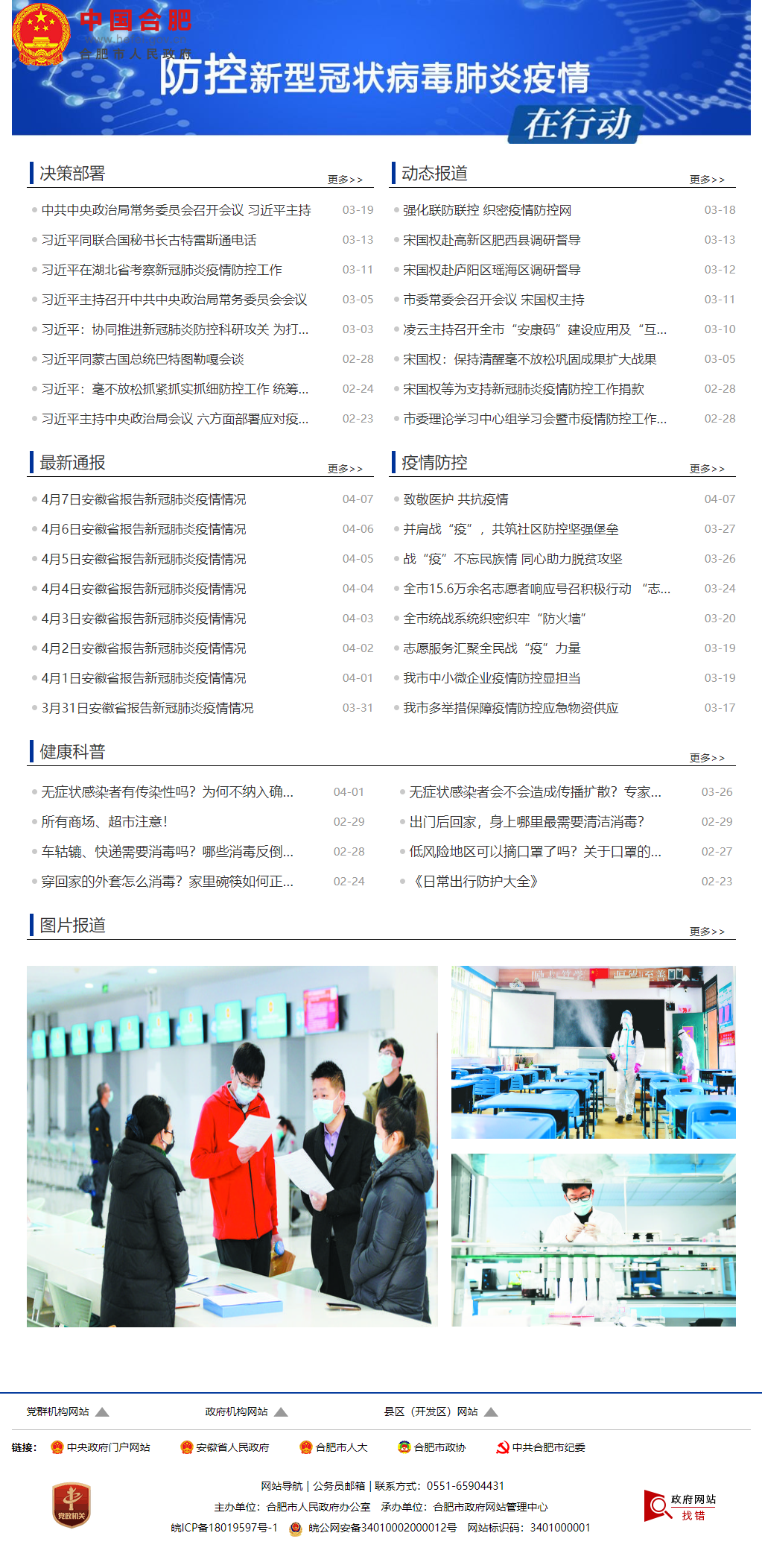
**

- **Hefei-Health department website**

**
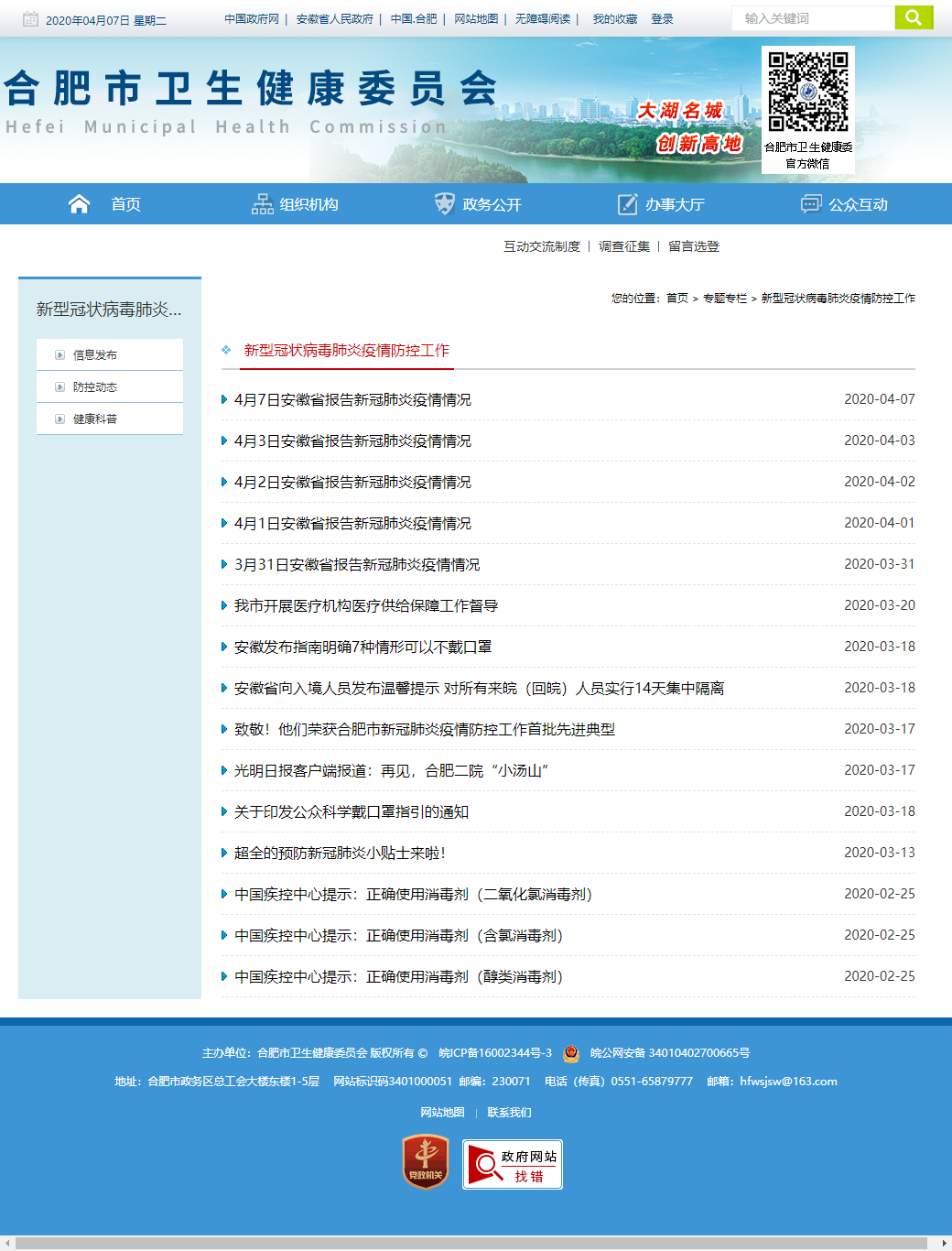
**

- **Fuzhou-Health department website**

**
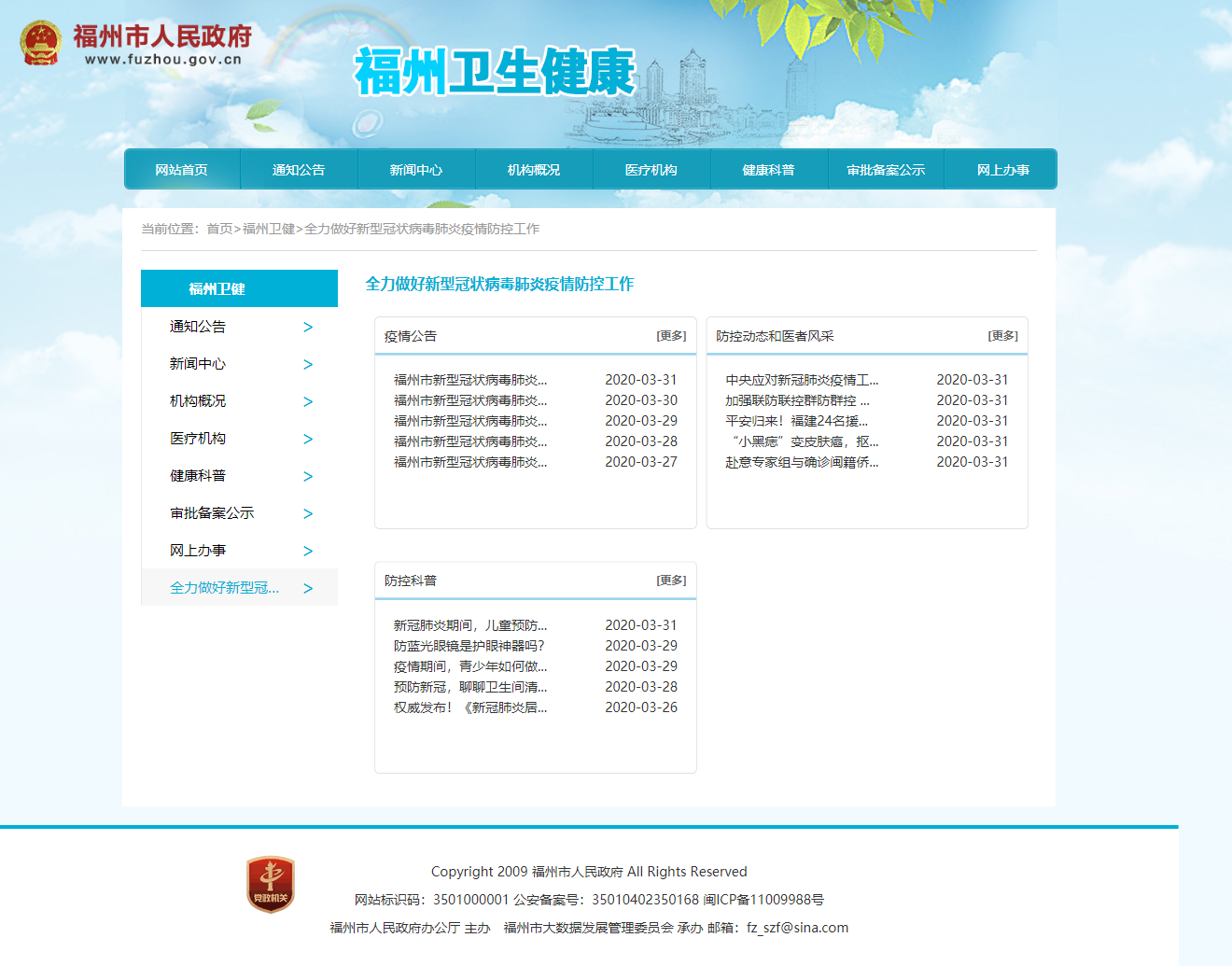
**

- **Nanchang-Municipality website**

**
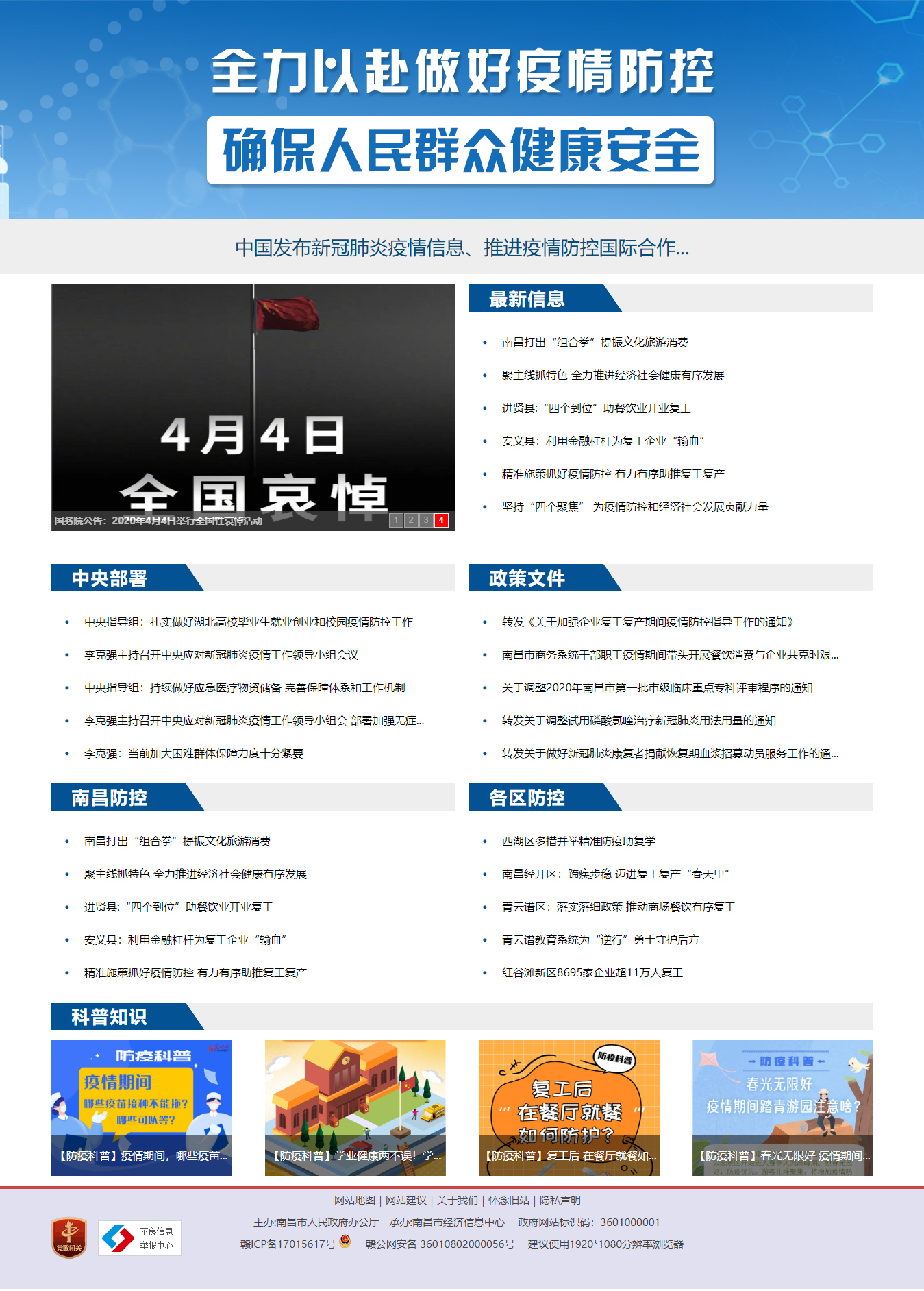
**

- **Ji'Nan-Health department website**

**
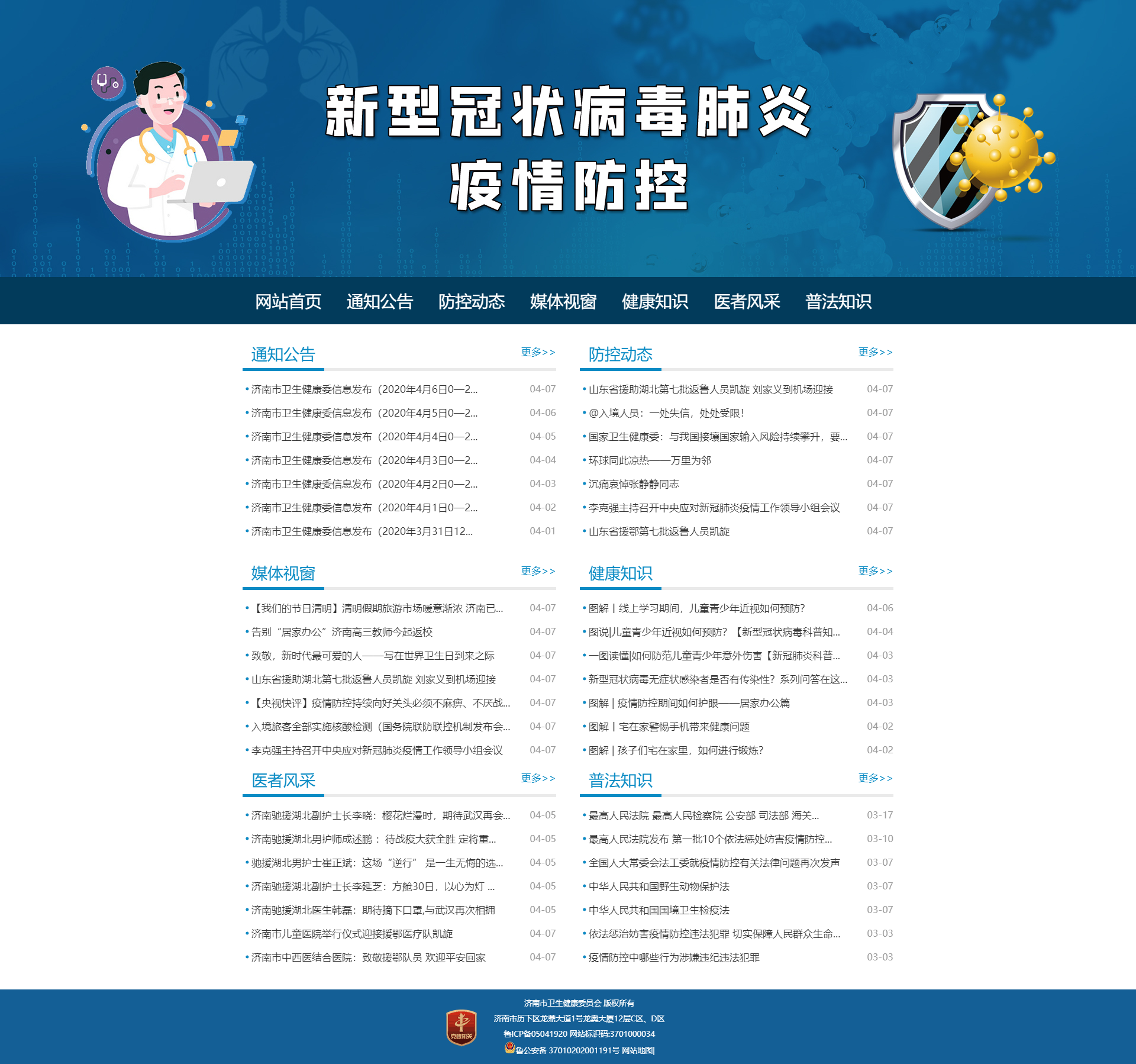
**

- **Zhengzhou-Health department website**

**
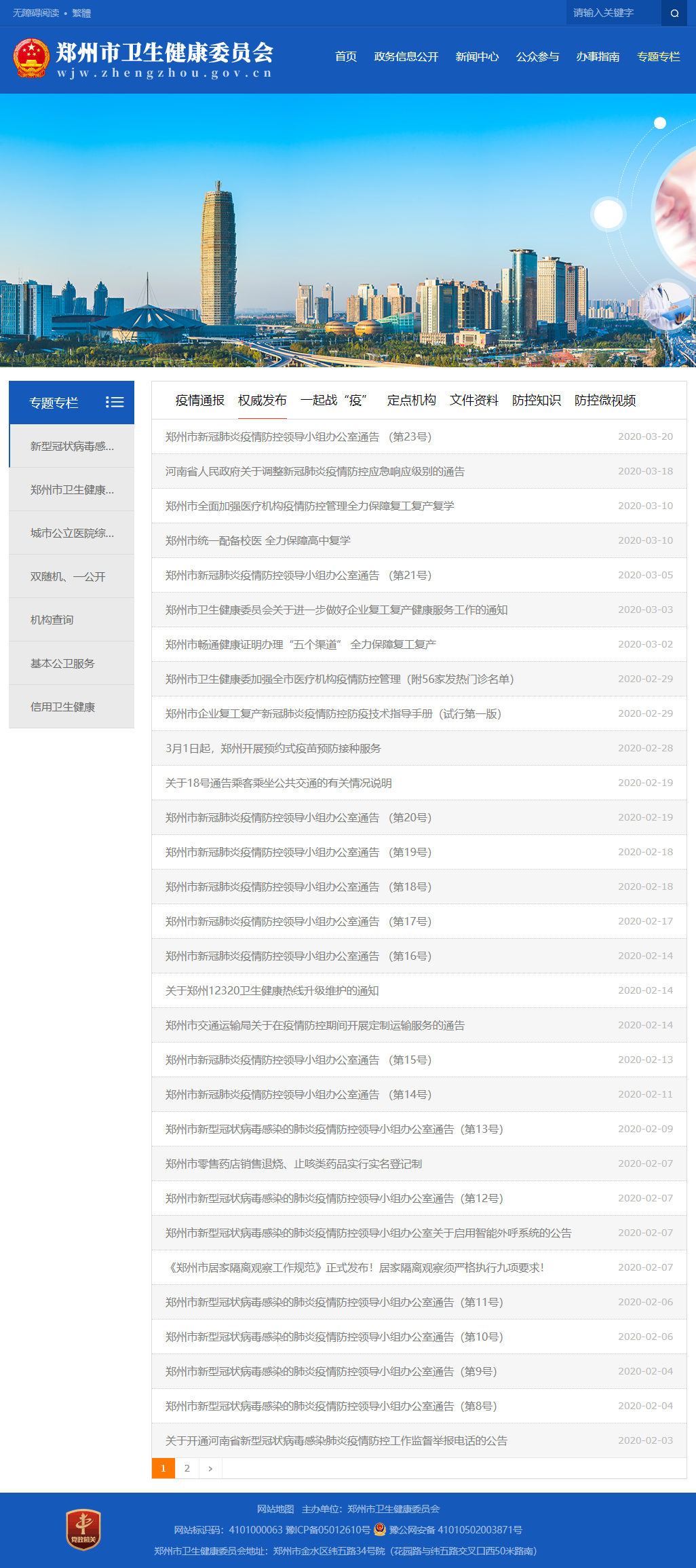
**

- **Wuhan-Health department website**

**
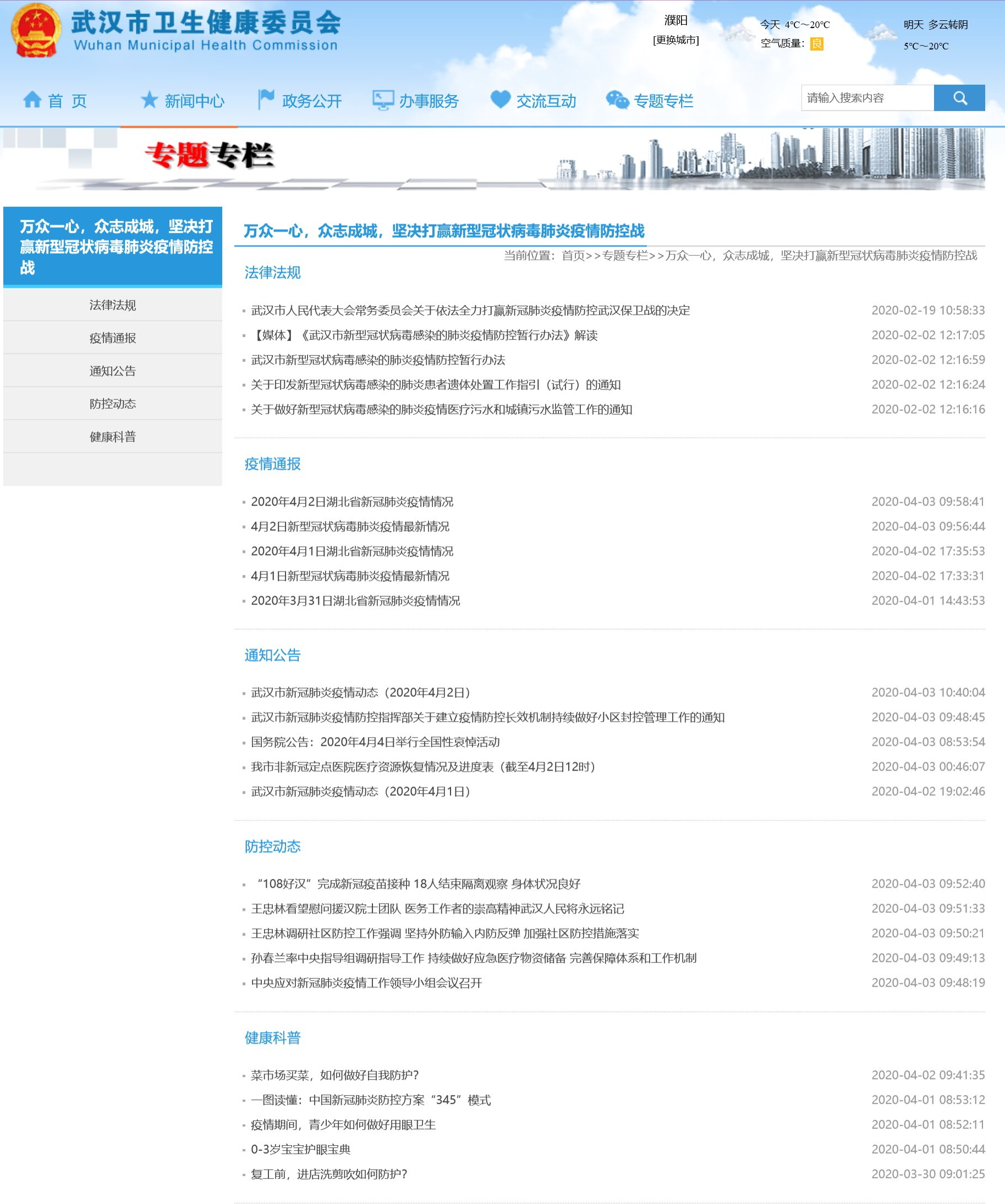
**

- **Changsha-Health department website**

**
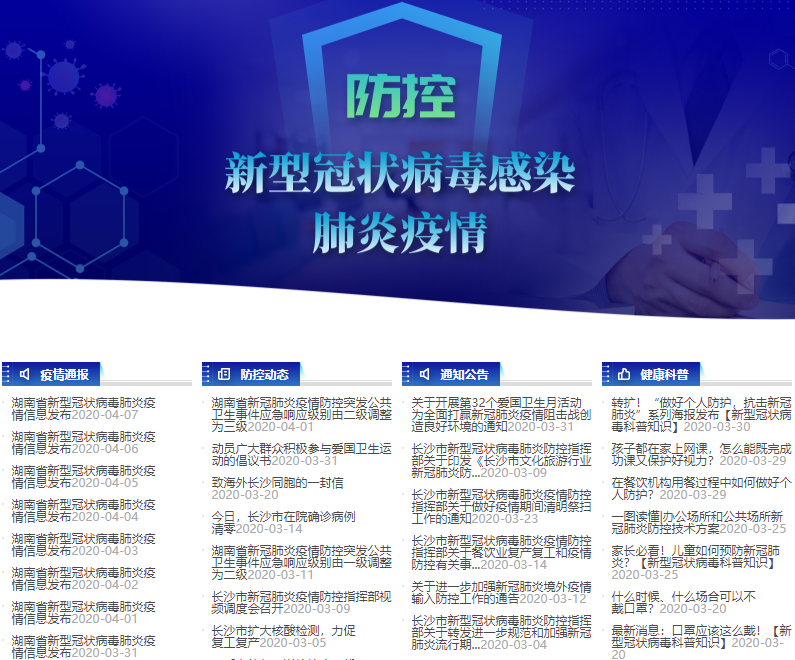
**

- **Guangzhou-Municipality**

**
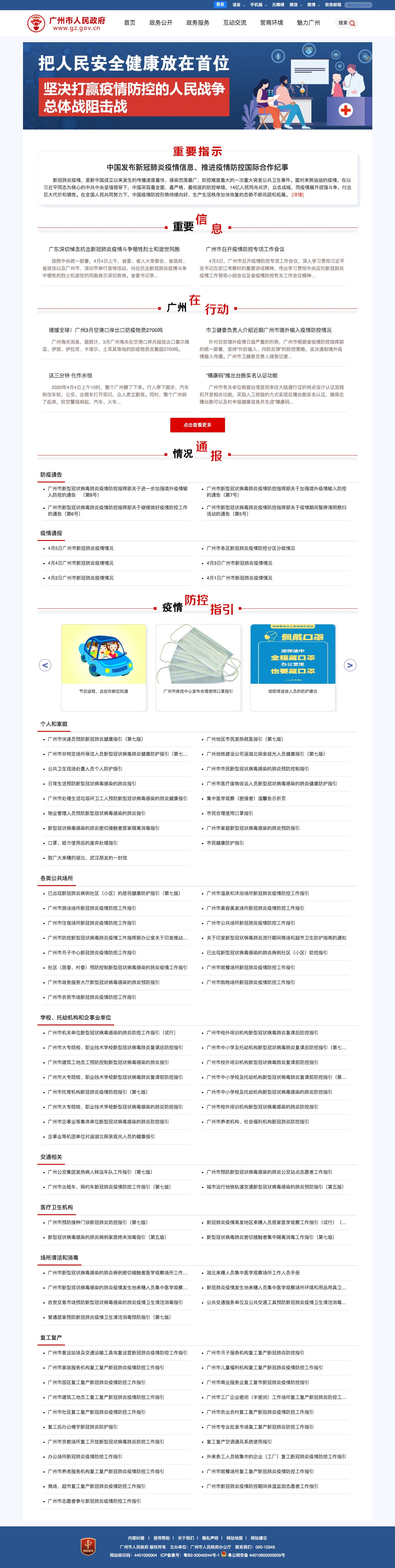
**

- **Guangzhou-Health department website**

**
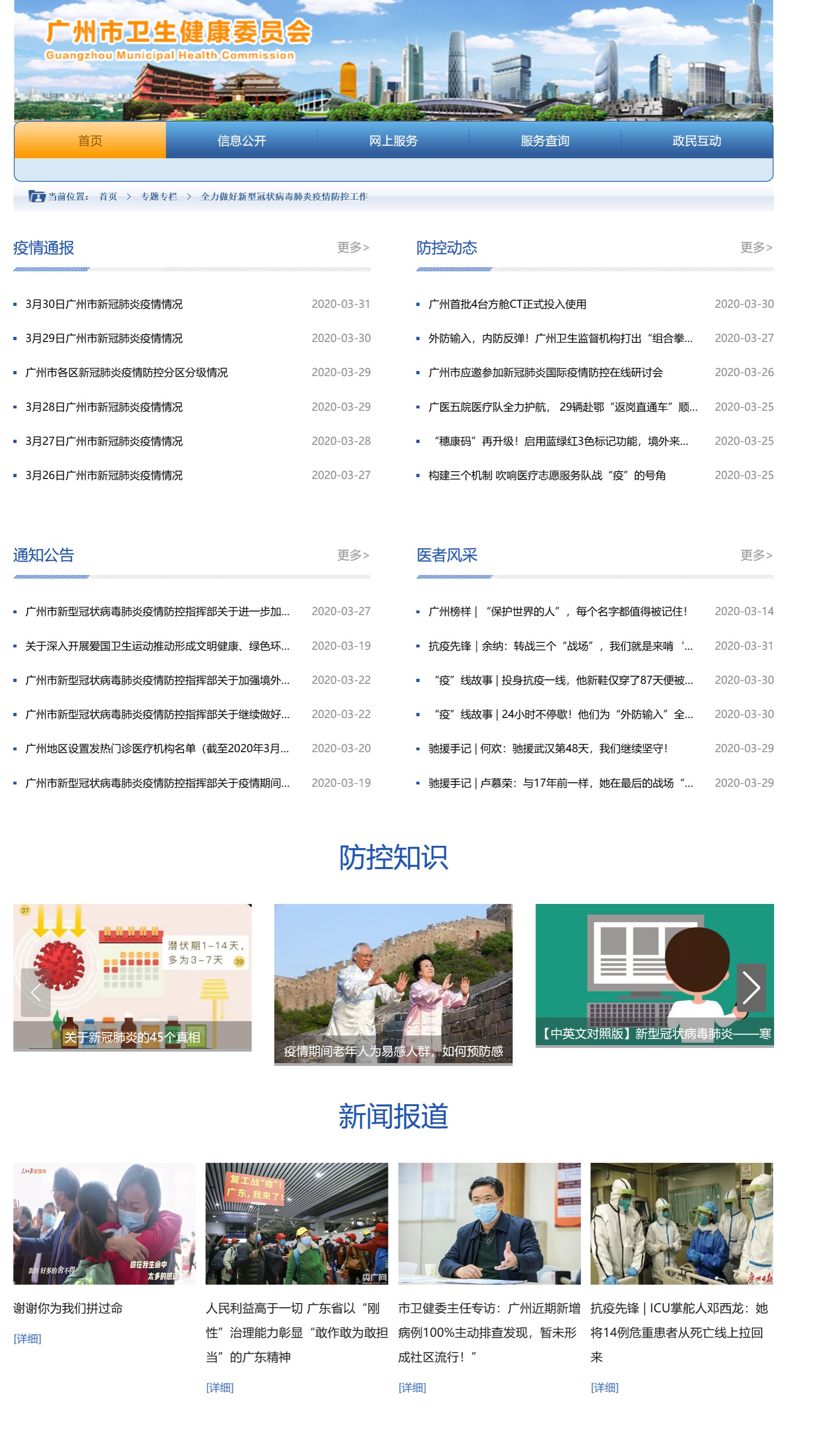
**

- **Nanning-Municipality website**

**
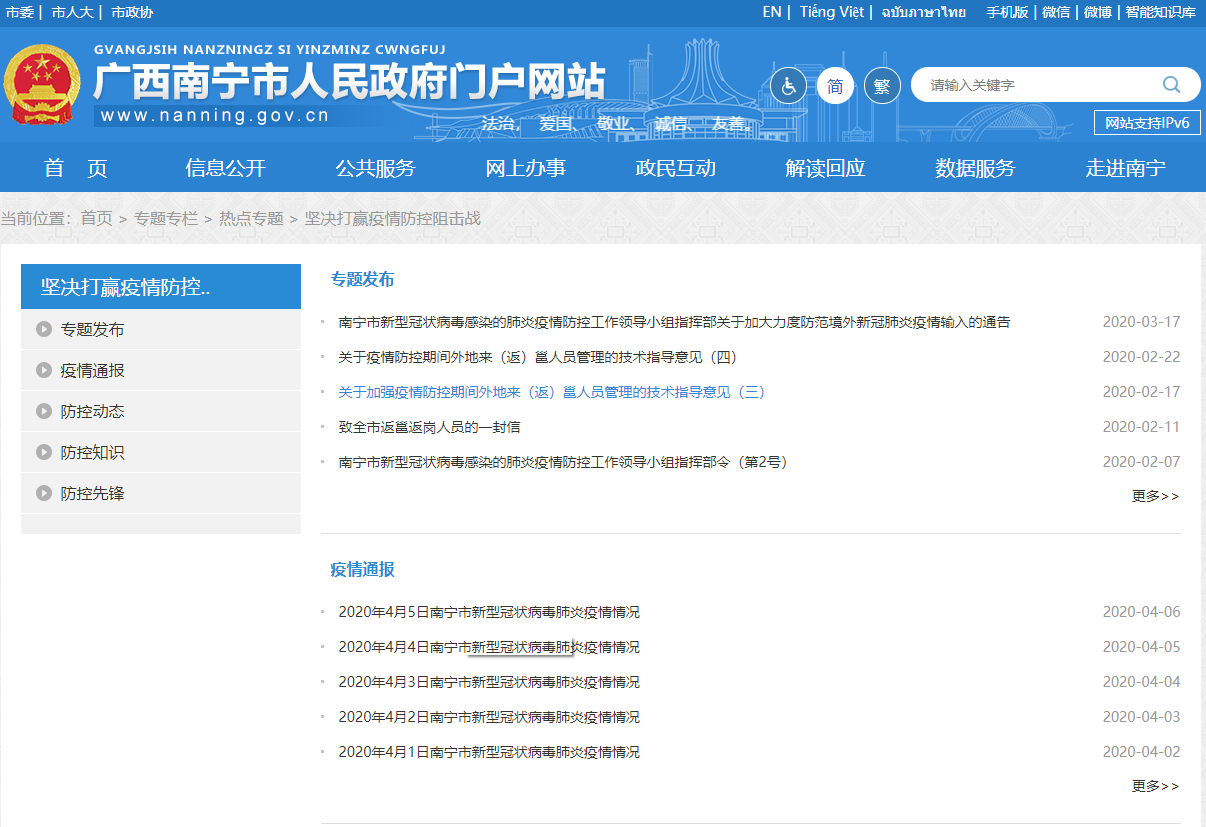
**

- **Nanning-Health department website**

**
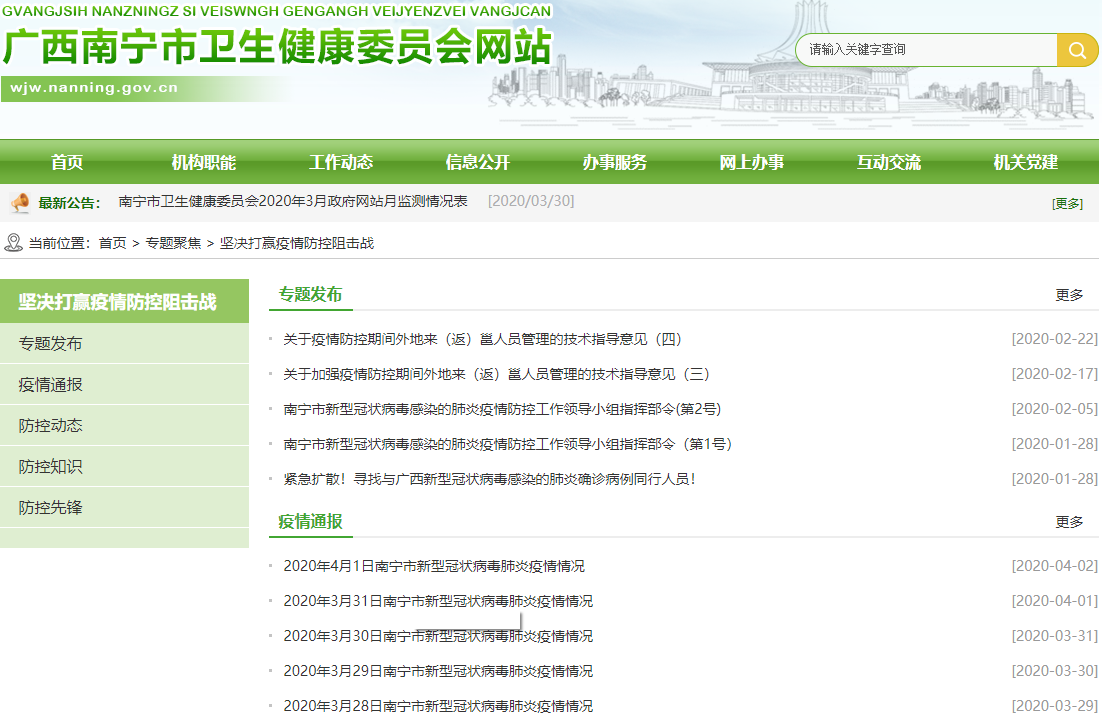
**

- **Haikou-Municipality website**

**
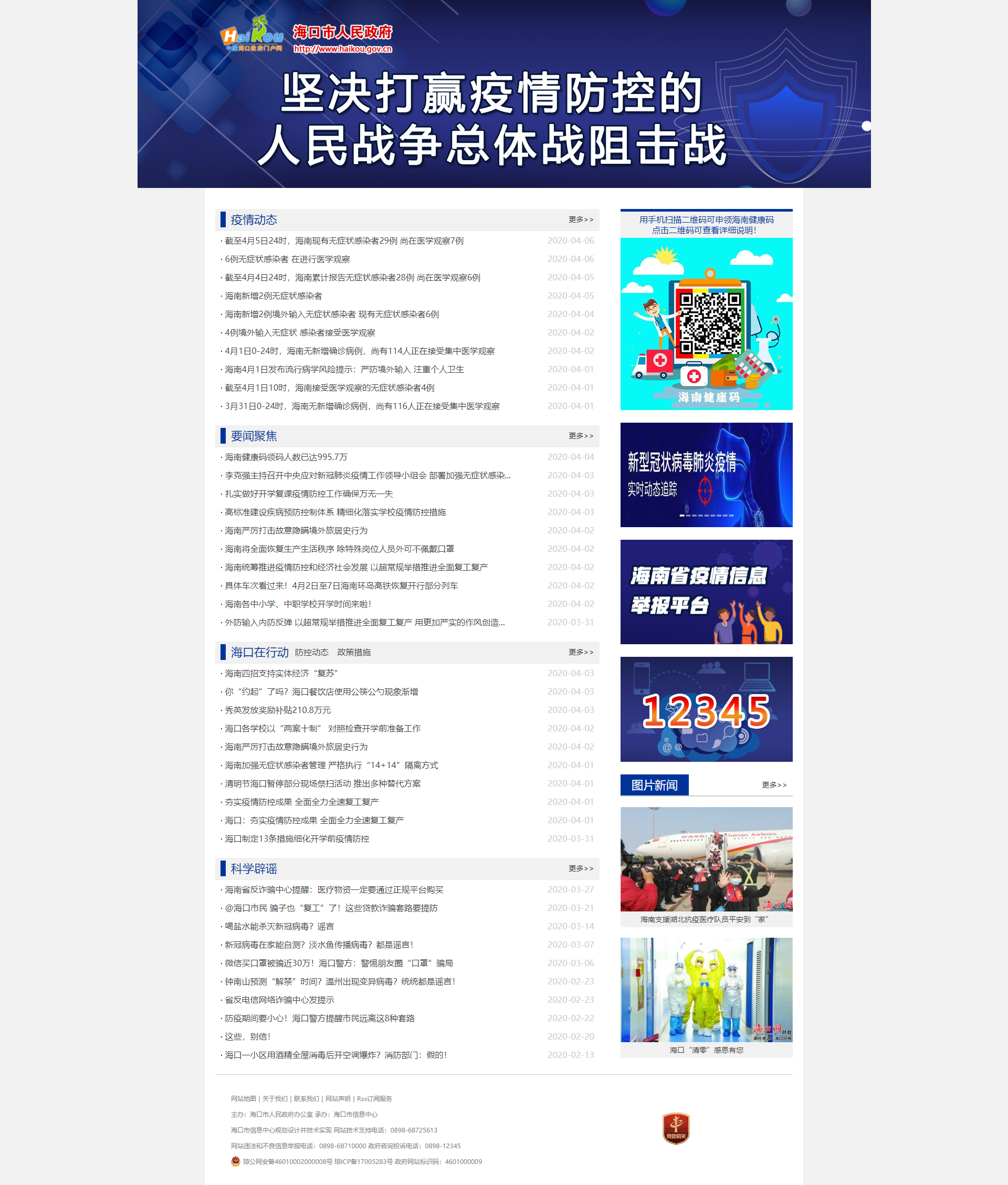
**

- **Chongqing-Municipality website**

**
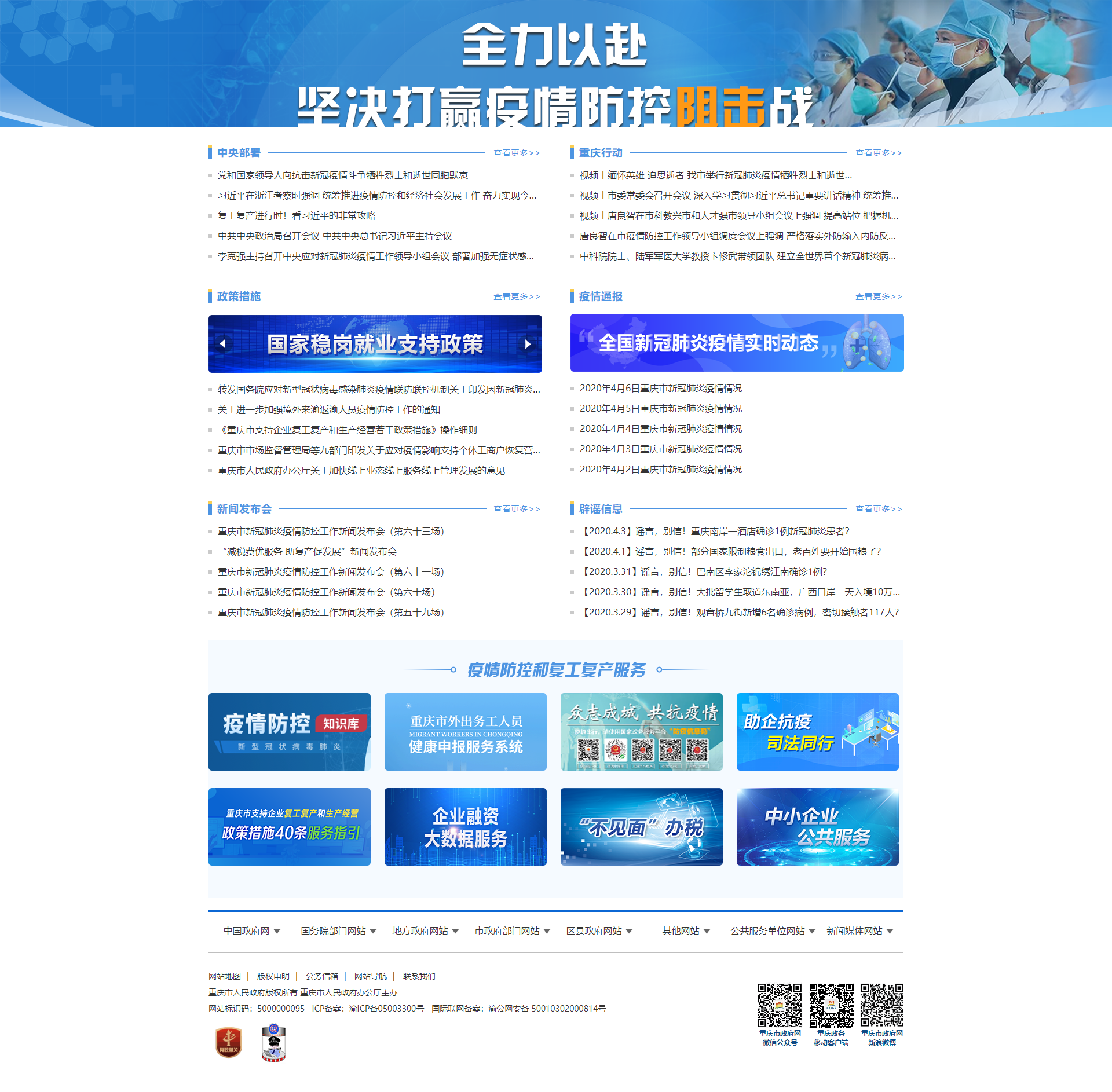
**

- **Chongqing-Health department website**

**
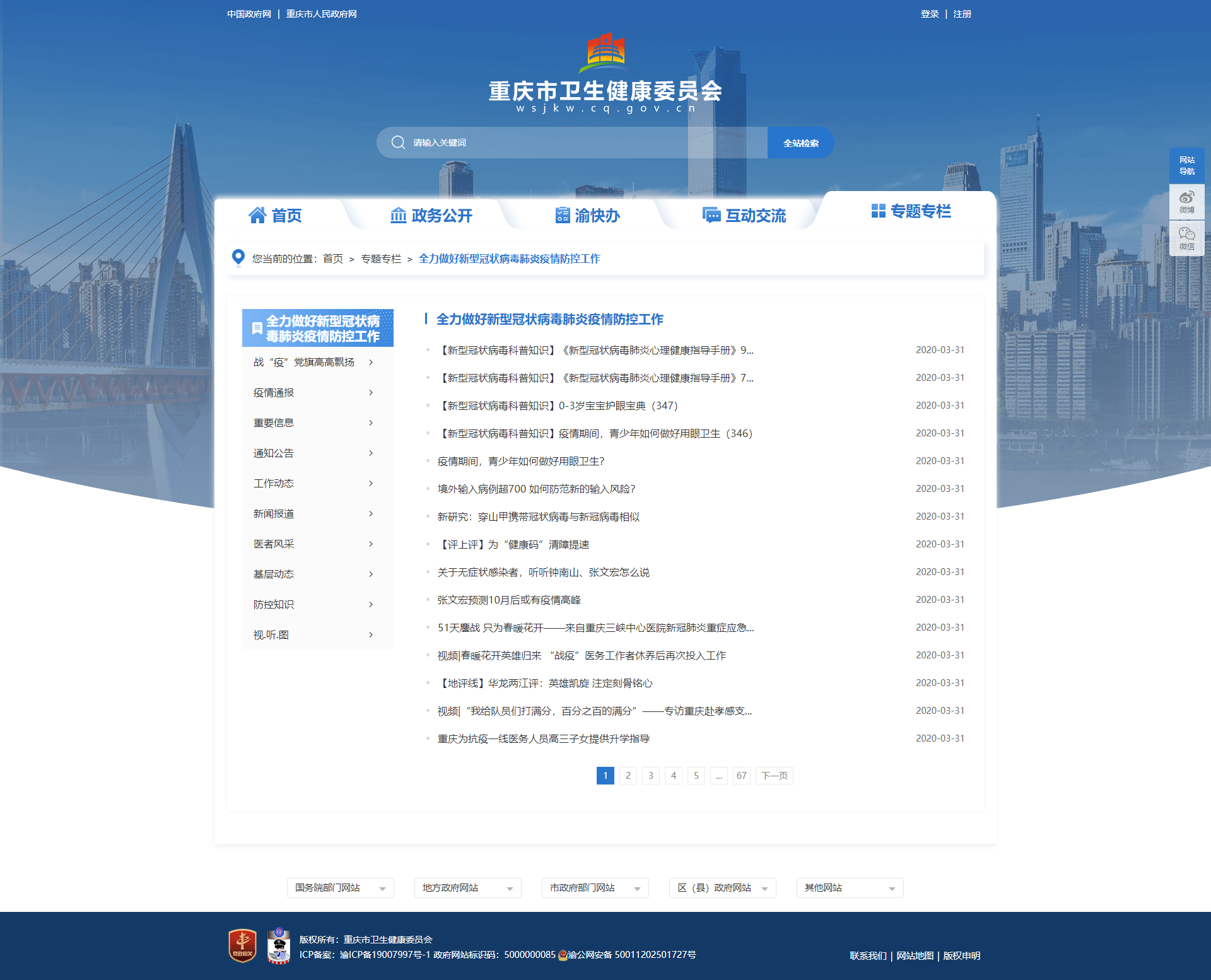
**

- **Chengdu-Municipality website**

**
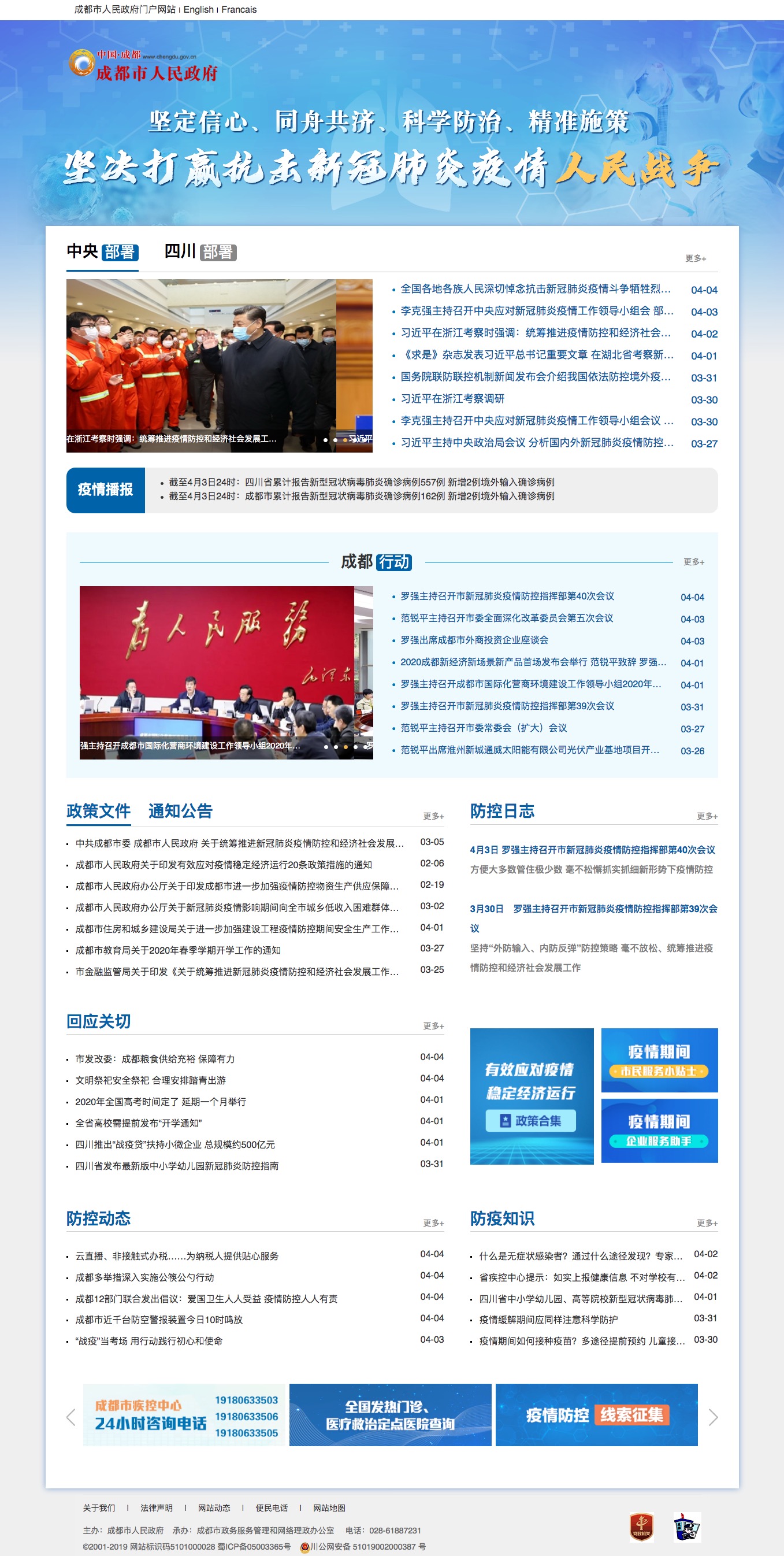
**

- **Chengdu-Health department website**

**
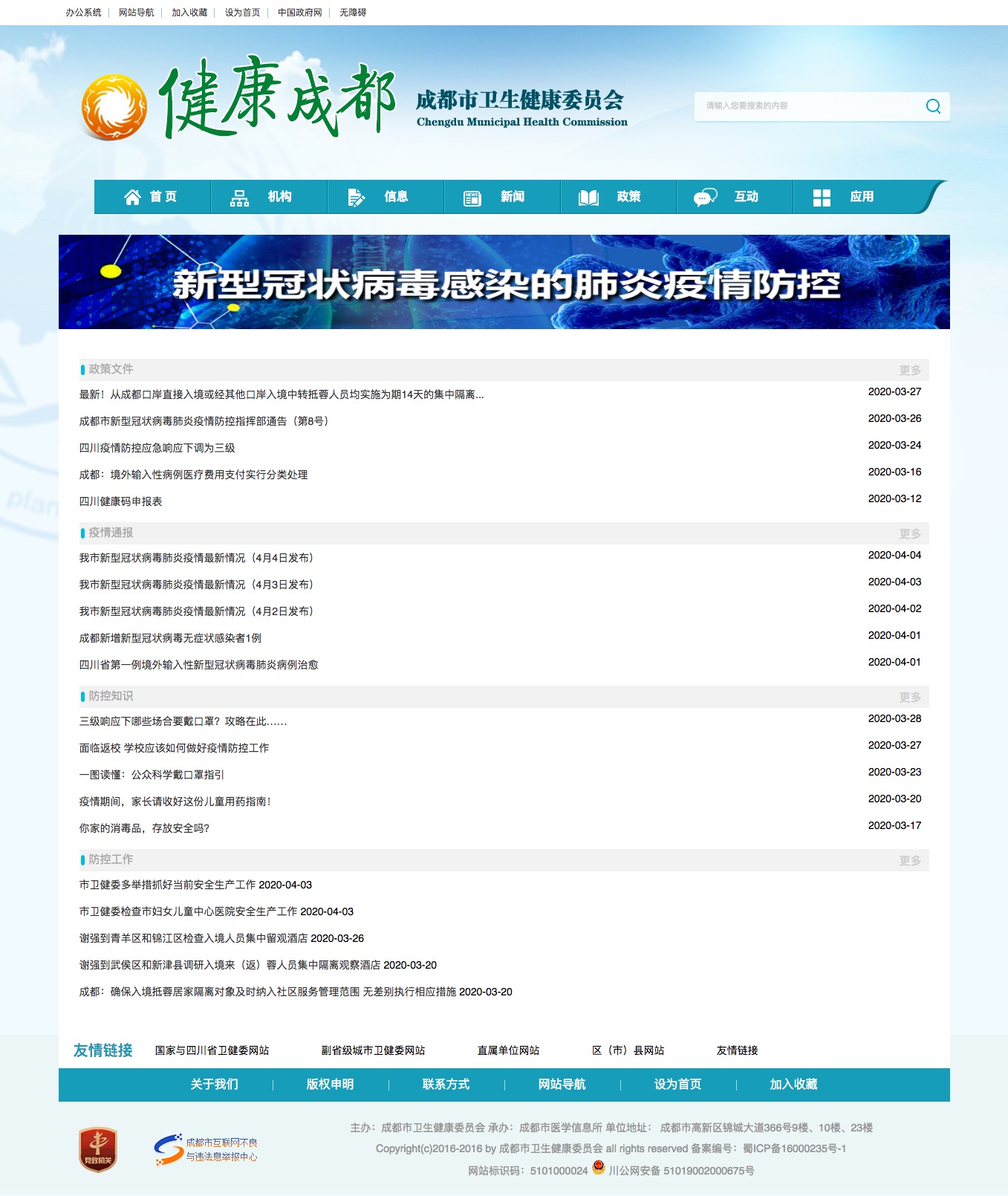
**

- **Guiyang-Health department website**

**
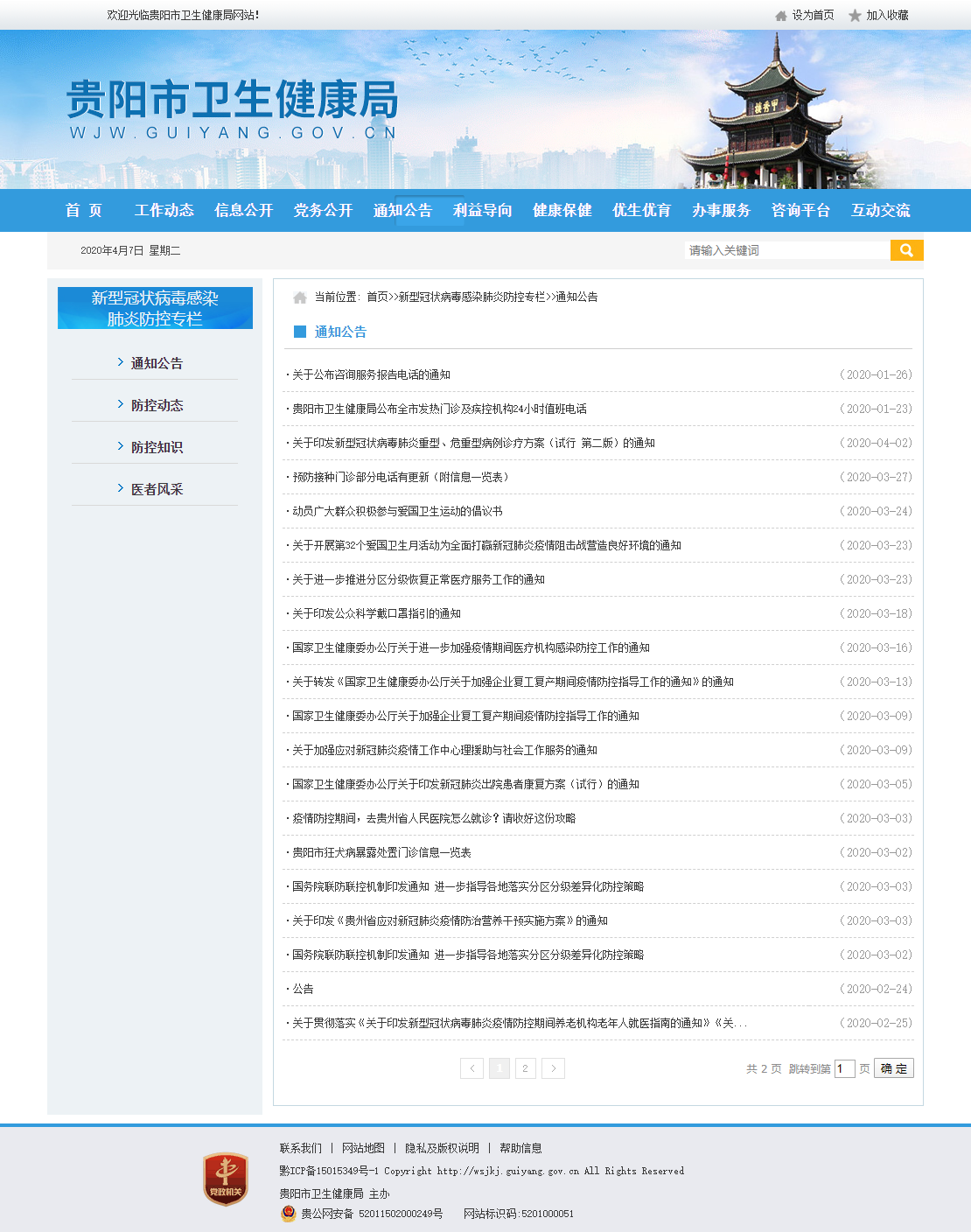
**

- **Lhasa-Municipality website**

**
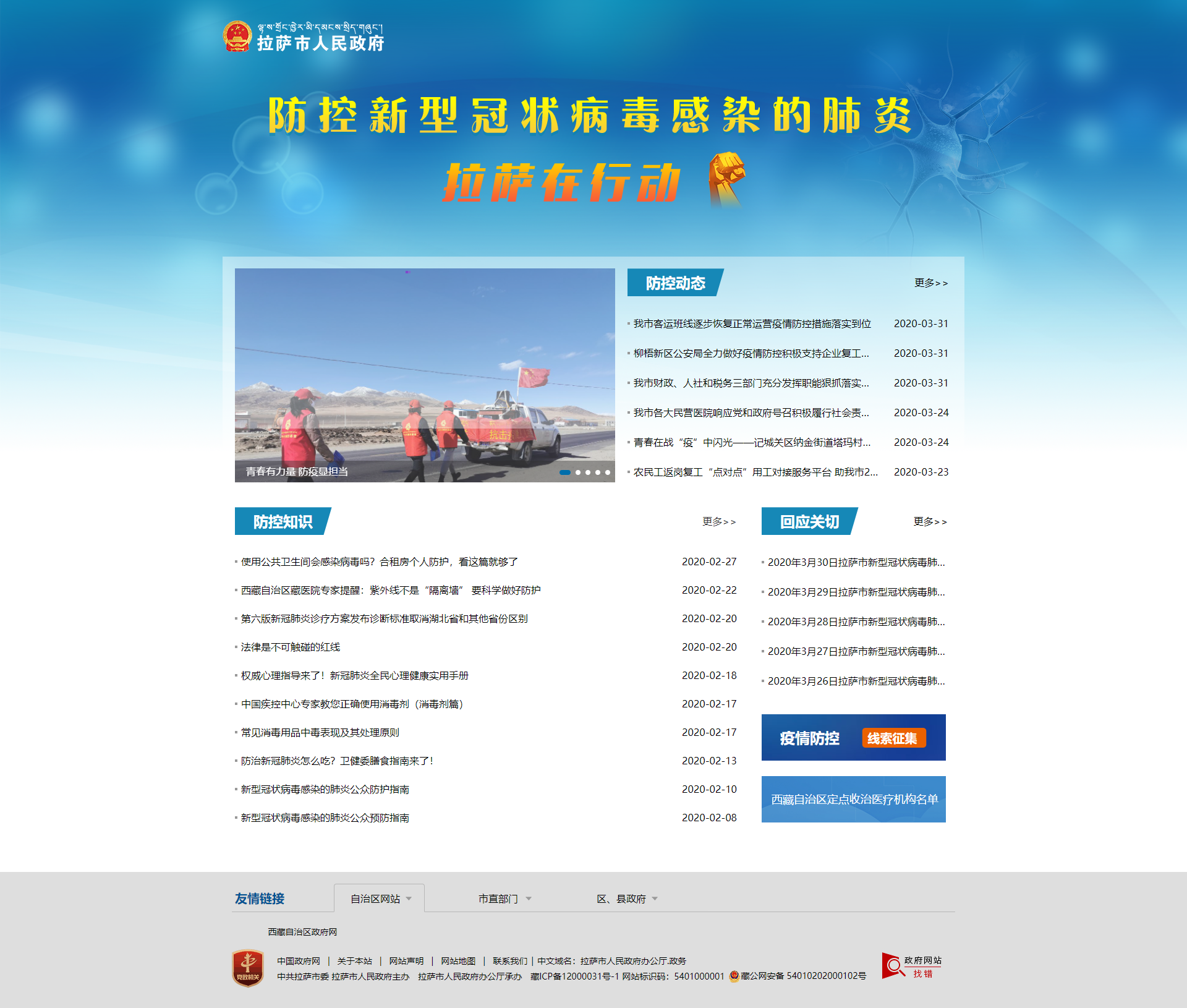
**

- **Xi'An-Health department website**

**
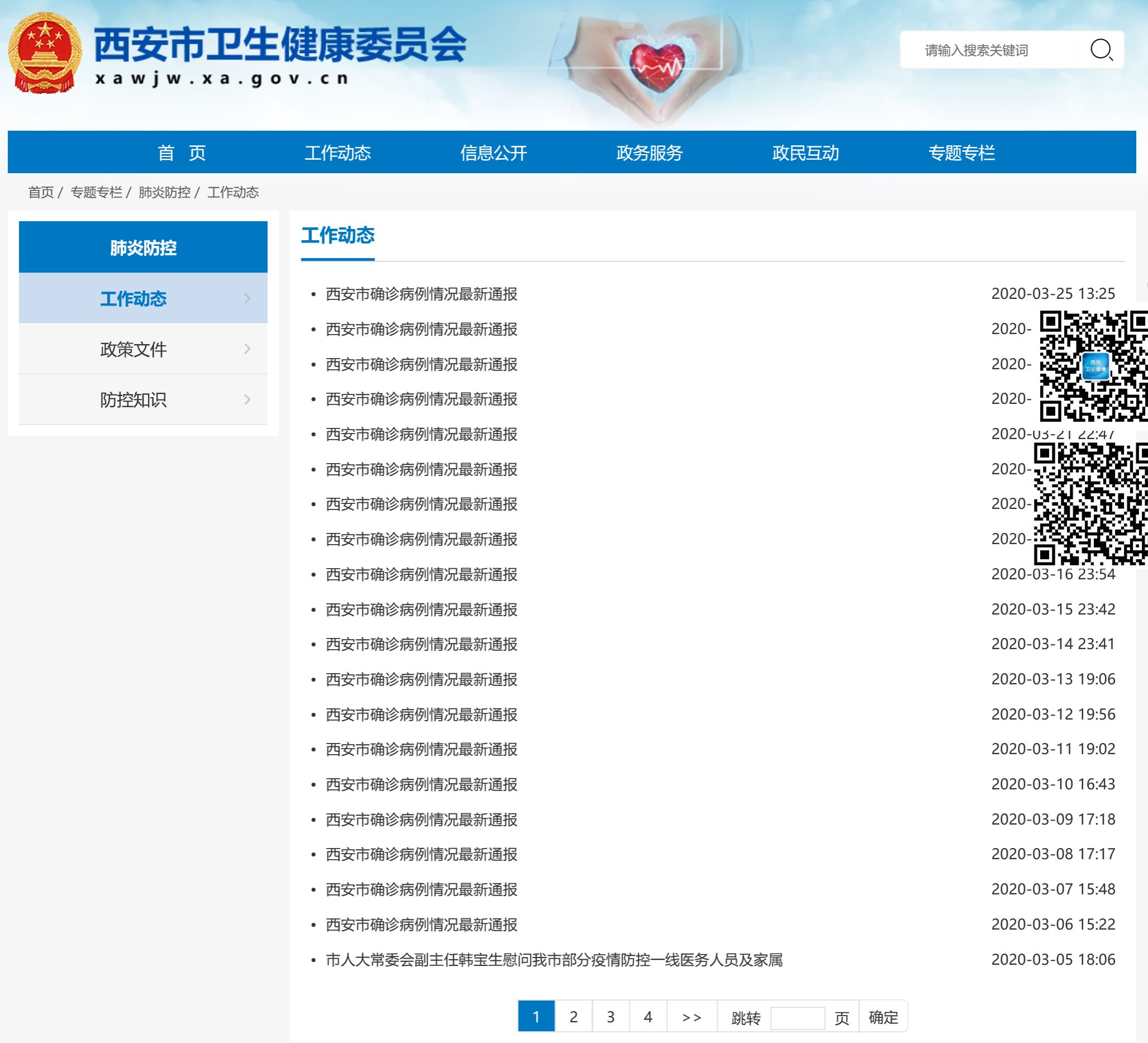
**

- **Lanzhou-Municipality website**

**
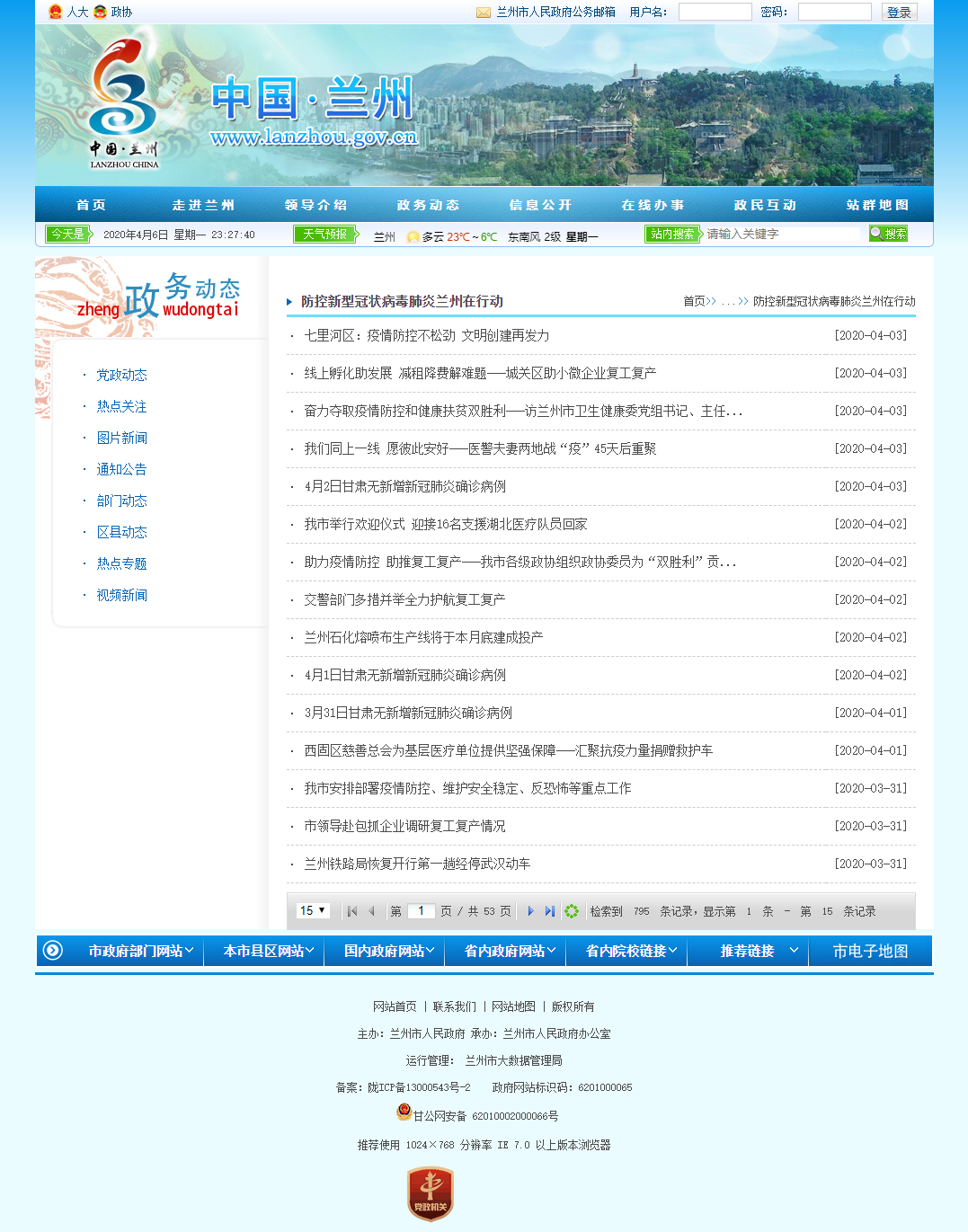
**

- **Xining-Health department website**

**
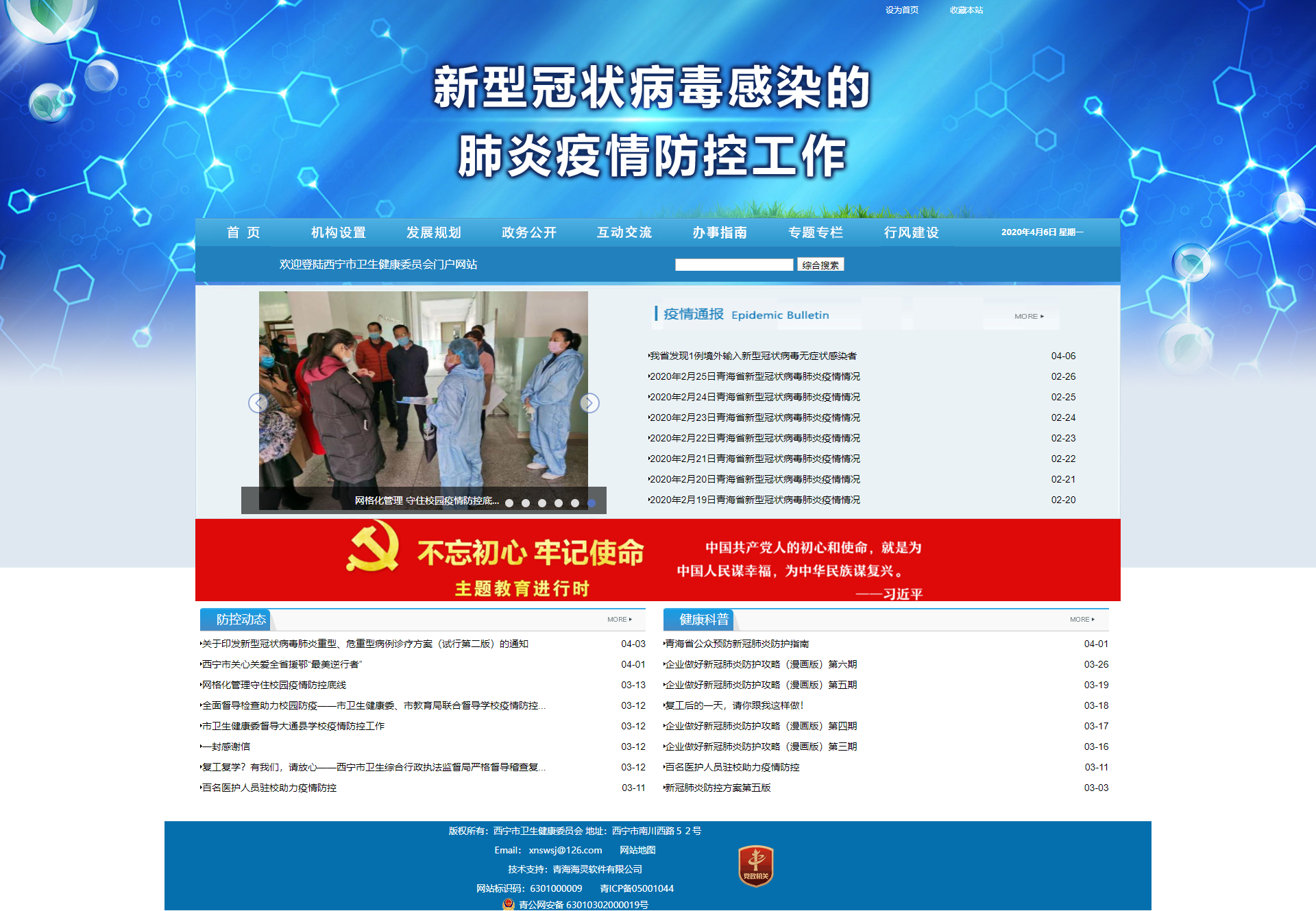
**

- **Yinchuan-Municipality website**

**
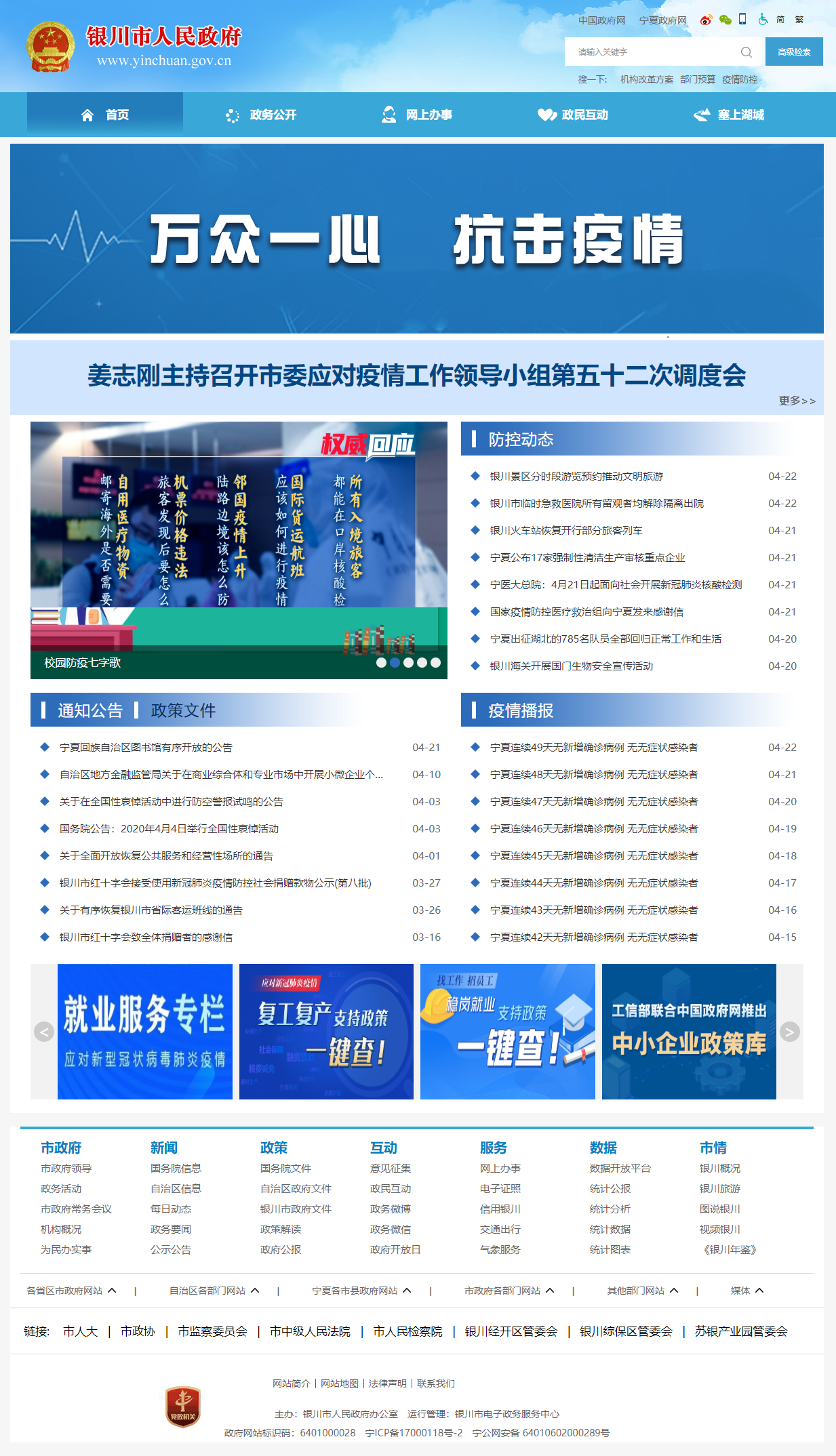
**

- **Urumqi-Municipality website**

**
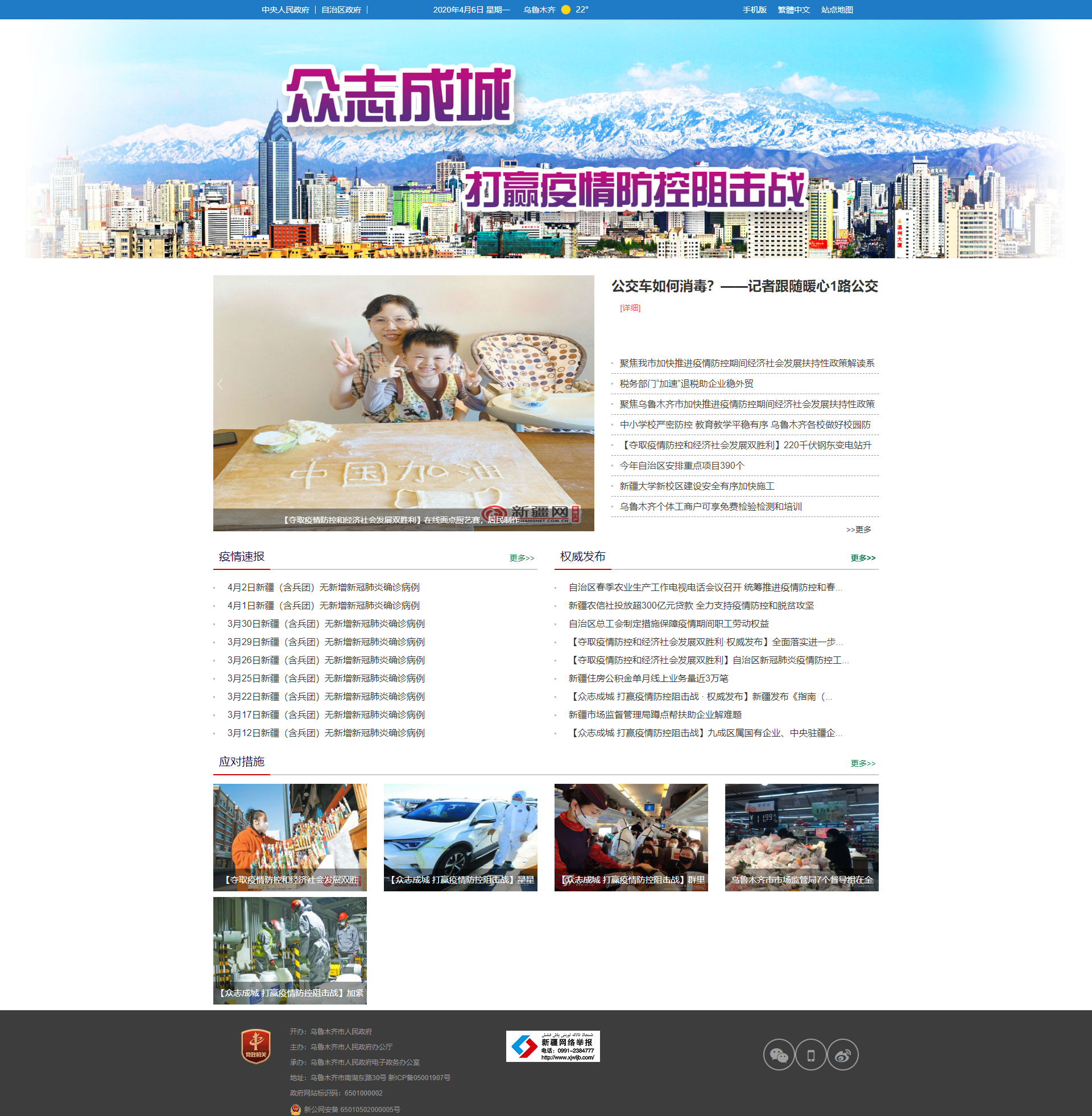
**
