# Supplementary material for: Information Disclosure During the COVID-19 Epidemic in China: City-Level Observational Study
Source: J Med Internet Res. 2020 Aug 27;22(8):e19572. doi: 10.2196/19572 (PMC7473703; doi:10.2196/19572)
Supplement: Multimedia Appendix 4 [file jmir_v22i8e19572_app4.docx]

| **Multimedia Appendix 4. Characteristics of cities with confirmed COVID-19 cases included in this analysis** | | | | | | | | | |
| --- | --- | --- | --- | --- | --- | --- | --- | --- | --- |
| **Type of city** | **City** | **COVID-19 webpage** | **Webpage base** | **Total population (10,000 persons)** | **Total confirmed cases by 18 March 2020** | **Number of hospitals** | **Number of licensed (assistant) doctors per 10,000 persons** | **Date of the first confirmed case** | **Date of the first case press briefing** |
| PC | Wuhan | √ | H-web | 884 | 50,005 | 398 | 48 | 2019-12-27 | 2019-12-31 |
|  | Guangzhou | √ | M-H | 928 | 359 | 255 | 58 | 2020-01-21 | 2020-01-22 |
|  | Changsha | √ | H-web | 729 | 242 | 232 | 42 | 2020-01-21 | 2020-01-22 |
|  | Nanchang | √ | M-web | 532 | 230 | 122 | 28 | 2020-01-22 | 2020-01-23 |
|  | Harbin | √ | H-web | 952 | 198 | 326 | 28 | 2020-01-23 | 2020-01-23 |
|  | Hangzhou | √ | M-web | 774 | 183 | 316 | 58 | 2020-01-21 | 2020-01-22 |
|  | Hefei | √ | M-H | 758 | 174 | 171 | 29 | 2020-01-22 | 2020-01-22 |
|  | Zhengzhou | √ | H-web | 864 | 157 | 246 | 49 | 2020-01-21 | 2020-01-21 |
|  | Chengdu | √ | M-H | 1,476 | 145 | 892 | 42 | 2020-01-21 | 2020-01-21 |
|  | Xi'An | √ | H-web | 987 | 120 | 343 | 34 | 2020-01-23 | 2020-01-23 |
|  | Nanjing | √ | M-web | 697 | 93 | 222 | 45 | 2020-01-23 | 2020-01-23 |
|  | Fuzhou | √ | H-web | 703 | 72 | 128 | 32 | 2020-01-22 | 2020-01-22 |
|  | Kunming | × | N/A | 572 | 53 | 322 | 50 | 2020-01-21 | 2020-01-22 |
|  | Ji'Nan | √ | H-web | 656 | 47 | 246 | 49 | 2020-01-24 | 2020-01-24 |
|  | Changchun | √ | M-web | 751 | 45 | 186 | 33 | 2020-01-20 | 2020-01-23 |
|  | Haikou | √ | M-web | 178 | 39 | 47 | 51 | 2020-01-22 | 2020-01-23 |
|  | Lanzhou | √ | M-web | 328 | 36 | 125 | 43 | 2020-01-23 | 2020-01-23 |
|  | Guiyang | √ | H-web | 418 | 36 | 192 | 43 | 2020-01-22 | 2020-01-22 |
|  | Shijiazhuang | √ | M-web | 982 | 29 | 256 | 38 | 2020-01-22 | 2020-01-23 |
|  | Shenyang | √ | M-web | 746 | 28 | 274 | 40 | 2020-01-22 | 2020-01-22 |
|  | Taiyuan | × | N/A | 377 | 20 | 163 | 61 | 2020-01-22 | 2020-01-22 |
|  | Xining | √ | H-web | 207 | 15 | 78 | 40 | 2020-01-25 | 2020-01-25 |
| AC | Nanning | √ | M-H | 771 | 55 | 120 | 32 | 2020-01-26 | 2020-01-26 |
|  | Yinchuan | √ | M-web | 193 | 36 | 86 | 50 | 2020-01-22 | 2020-01-22 |
|  | Urumqi | √ | M-web | 222 | 23 | 130 | 68 | 2020-01-23 | 2020-01-23 |
|  | Hohhot | √ | M-web | 246 | 7 | 108 | 44 | 2020-01-28 | 2020-01-28 |
|  | Lhasa | √ | M-web | 55 | 1 | 29 | 52 | 2020-01-30 | 2020-01-30 |
| MC | Chongqing | √ | M-H | 3,404 | 576 | 800 | 22 | 2020-01-21 | 2020-01-21 |
|  | Beijing | √ | M-H | 1,376 | 479 | 648 | 73 | 2020-01-19 | 2020-01-20 |
|  | Shanghai | √ | M-H | 1,462 | 363 | 358 | 49 | 2020-01-20 | 2020-01-20 |
|  | Tianjin | √ | M-H | 1,082 | 136 | 420 | 40 | 2020-01-21 | 2020-01-21 |
| PC = Provincial capital, AC = Autonomous region capital, MC = Municipalities administered by the central government., COVID-19 = Coronavirus disease of 2019, M-Web = Municipality website. H-Web = Health department website. M-H = Both municipality and health department websites. N/A = Not applicable. | | | | | | | | | |
| Data on total population, number of hospitals, number of licensed (assistant) doctors per 10,000 persons as of 2018; all come from the China Statistical Database. (<http://data.stats.gov.cn>)  Chinese scientists identified the pathogen as a novel coronavirus on January 7, 2020. The first case with symptoms of the novel coronavirus in Wuhan was identified and reported to the authorities on December 27, 2019, by Jixian Zhang, the director of the Department of Respiratory and Critical Care Medicine of Hubei Provincial Hospital of Integrated Chinese and Western Medicine. China’s National Health Commission incorporated COVID-19 as a notifiable disease in the National Infectious Disease Law and the Frontier Health and Quarantine Law on 20 January 20, 2020. | | | | | | | | | |
