# Supplementary material for: Information Disclosure During the COVID-19 Epidemic in China: City-Level Observational Study
Source: J Med Internet Res. 2020 Aug 27;22(8):e19572. doi: 10.2196/19572 (PMC7473703; doi:10.2196/19572)
Supplement: Multimedia Appendix 5 [file jmir_v22i8e19572_app5.docx]

| **Multimedia Appendix 5. Percentage of cities with content categories covered on official COVID-19 websites, March 2020** | | | | |
| --- | --- | --- | --- | --- |
| **Categories** | **Total (N=29), n (%)** | **PC (n=20), n (%)** | **AC (n=5), n (%)** | **MC (n=4), n (%)** |
| News updates | 28(96.6) | 19(95.0) | 5(100.0) | 4(100.0) |
| Epidemic surveillance | 25(86.2) | 16(80.0) | 5(100.0) | 4(100.0) |
| Advice for public | 25(86.2) | 18(90.0) | 3(60.0) | 4(100.0) |
| Authority announcement | 21(72.4) | 13(65.0) | 4(80.0) | 4(100.0) |
| Local action | 15(51.7) | 9(45.0) | 3(60.0) | 3(75.0) |
| Misinformation clarification | 2(6.9) | 1(5.0) | 0(0.0) | 1(25.0) |
| Frequently Asked Questions (FAQ) | 0(0.0) | 0(0.0) | 0(0.0) | 0(0.0) |
| Data are n (%) unless otherwise specified. PC = Provincial capitals, AC = Capitals of autonomous regions, MC = Municipalities administered by the central government. | | | | |
