# Supplementary material for: Information Disclosure During the COVID-19 Epidemic in China: City-Level Observational Study
Source: J Med Internet Res. 2020 Aug 27;22(8):e19572. doi: 10.2196/19572 (PMC7473703; doi:10.2196/19572)
Supplement: Multimedia Appendix 6 [file jmir_v22i8e19572_app6.docx]

| **Multimedia Appendix 6. Percentage of cities with key indicators revealed in epidemic surveillance summaries, as of March 18, 2020** | | | | |
| --- | --- | --- | --- | --- |
| **Indicators** | **Total(N=25), n (%)** | **PC(n=16), n (%)** | **AC(n=5), n (%)** | **MC(n=4), n (%)** |
| Cumulative confirmed cases | 24(96.0) | 16(100.0) | 4(80.0) | 4(100.0) |
| Daily confirmed cases | 22(88.0) | 14(87.5) | 4(80.0) | 4(100.0) |
| Cumulative discharged cases | 18(72.0) | 11(68.8) | 3(60.0) | 4(100.0) |
| Active Cases | 14(56.0) | 9(56.3) | 2(40.0) | 3(75.0) |
| Cumulative deceased cases | 12(48.0) | 7(43.8) | 1(20.0) | 4(100.0) |
| Daily discharged cases | 11(44.0) | 7(43.8) | 1(20.0) | 3(75.0) |
| Hospitalized critical cases | 8(32.0) | 4(25.0) | 1(20.0) | 3(75.0) |
| Daily suspected cases | 8(32.0) | 4(25.0) | 4(80.0) | 0(0.0) |
| Daily deceased cases | 5(20.0) | 2(12.5) | 1(20.0) | 2(50.0) |
| Hospitalized stable cases | 4(16.0) | 2(12.5) | 0(0.0) | 2(50.0) |
| Data are n (%) unless otherwise specified. PC = Provincial capitals, AC = Capitals of autonomous regions, MC = Municipalities administered by the central government. | | | | |
