# Supplementary material for: Information Disclosure During the COVID-19 Epidemic in China: City-Level Observational Study
Source: J Med Internet Res. 2020 Aug 27;22(8):e19572. doi: 10.2196/19572 (PMC7473703; doi:10.2196/19572)
Supplement: Multimedia Appendix 7 [file jmir_v22i8e19572_app7.docx]

| **Multimedia Appendix 7. Percentage of cities with key facts disclosed from the latest confirmed case reports, as of March 18, 2020** | | | | |
| --- | --- | --- | --- | --- |
| **Categories** | **Total(N=18), n (%)** | **PC(n=12), n (%)** | **AC(n=3), n (%)** | **MC(n=3), n (%)** |
| Gender | 16(88.9) | 11(91.7) | 3(100.0) | 2(66.7) |
| Date of confirmation | 15(83.3) | 11(91.7) | 2(66.7) | 2(66.7) |
| Age | 15(83.3) | 10(83.3) | 3(100.0) | 2(66.7) |
| Places visited | 13(72.2) | 8(66.7) | 2(66.7) | 3(100.0) |
| Contact tracing | 11(61.1) | 6(50.0) | 2(66.7) | 3(100.0) |
| Patient status | 11(61.1) | 7(58.3) | 2(66.7) | 2(66.7) |
| Name of hospital admitted | 10(55.6) | 7(58.3) | 2(66.7) | 1(33.3) |
| Imported/Local | 9(50.0) | 5(41.7) | 1(33.3) | 3(100.0) |
| Nationality | 9(50.0) | 4(33.3) | 2(66.7) | 3(100.0) |
| Residence | 9(50.0) | 8(66.7) | 1(33.3) | 0(0.0) |
| Anonymous ID | 2(11.1) | 1(8.3) | 0(0.0) | 1(33.3) |
| Data are n (%) unless otherwise specified. PC = Provincial capitals, AC = Capitals of autonomous regions, MC = Municipalities administered by the central government. | | | | |
